# Quaternary arrangement of an active, native group II intron ribonucleoprotein complex revealed by small-angle X-ray scattering

**DOI:** 10.1093/nar/gku140

**Published:** 2014-02-24

**Authors:** Kushol Gupta, Lydia M. Contreras, Dorie Smith, Guosheng Qu, Tao Huang, Lynn A. Spruce, Steven H. Seeholzer, Marlene Belfort, Gregory D. Van Duyne

**Affiliations:** ^1^Department of Biochemistry & Biophysics, Perelman School of Medicine, University of Pennsylvania, Philadelphia, PA 19104-6059, USA, ^2^Department of Chemical Engineering, University of Texas at Austin, Austin, TX 78712, USA, ^3^Wadsworth Center, NYS Department of Health, Albany, NY 12201, USA, ^4^Department of Biological Sciences and RNA Institute, University at Albany, State University of New York, Albany, NY 12222, USA, ^5^SUNY Downstate Medical Center, University Hospital, Brooklyn, NY 11203, USA and ^6^Children's Hospital of Philadelphia Research Institute, Philadelphia, PA 19104, USA

## Abstract

The stable ribonucleoprotein (RNP) complex formed between the *Lactococcus lactis* group II intron and its self-encoded LtrA protein is essential for the intron’s genetic mobility. In this study, we report the biochemical, compositional, hydrodynamic and structural properties of active group II intron RNP particles (+A) isolated from its native host using a novel purification scheme. We employed small-angle X-ray scattering to determine the structural properties of these particles as they exist in solution. Using sucrose as a contrasting agent, we derived a two-phase quaternary model of the protein–RNA complex. This approach revealed that the spatial properties of the complex are largely defined by the RNA component, with the protein dimer located near the center of mass. A transfer RNA fusion engineered into domain II of the intron provided a distinct landmark consistent with this interpretation. Comparison of the derived +A RNP shape with that of the previously reported precursor intron (ΔA) particle extends previous findings that the loosely packed precursor RNP undergoes a dramatic conformational change as it compacts into its active form. Our results provide insights into the quaternary arrangement of these RNP complexes in solution, an important step to understanding the transition of the group II intron from the precursor to a species fully active for DNA invasion.

## INTRODUCTION

**Figure 1.**
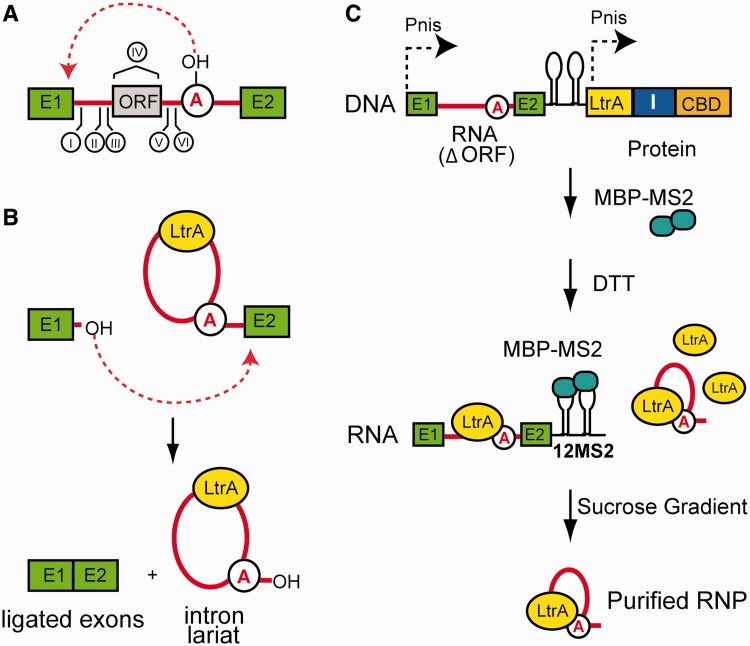
Domain structure, activity and purification of native group II introns. (**A**) Domain structure of a group II intron. Rendered as squares are the exons (E1 and E2) flanking the group II intron sequence. Denoted with Roman numerals are the six structural domains of the intron. Domain IV contains an ORF that encodes an IEP, also referred to as LtrA. Domain VI contains the catalytic adenosine nucleophile (circled) required for splicing. (**B**) Splicing reaction carried out by group II introns. Group II introns are excised from flanking exons through a two-step process that is catalyzed by the intron RNA. (**C**) Purification strategy. Lysates from nisin-induced cells are passed over a chitin resin column to capture LtrA–intein–CBD protein fusion and associated RNA, via a C-terminal chitin-binding domain in the LtrA fusion. I = intein. To facilitate separation (see below), MBP-MS2 (turquoise balls) is added to bind the MS2 tag in the precursor at the 3′ exon (black lollipops). Cleavage of LtrA from the intein with DTT) releases the RNP from the chitin column in both active and precursor forms. Assuming a 1:1 stoichiometry between MBP-MS2 protein (∼50 kD) and its RNA-binding site (21), addition of the MS2 binding protein further increases the size differential between the 12xMS2-containing precursor (∼1.4 MDa) and the spliced lariats form (∼430 kD) of the RNP. This large size differential allows for successful final isolation of the active RNP from the precursor species by sedimentation, using a sucrose cushion gradient.

Group II introns are catalytic RNAs found in all three domains of life (1,2). This group of mobile genetic elements provides an important model system to understand RNA catalysis because of the diversity of the chemical reactions performed in partnership with a helper protein (3). Interest in this class of introns also derives from their inferred ancestral relationship to nuclear spliceosomal introns (4–9) and retrotransposons (10) and their evolutionary impact on host genomes. Mobile group II introns specify intron-encoded proteins (IEPs) with reverse transcriptase and maturase activity, which facilitates the proper folding of the intron precursor RNA (Figure 1A) (11,12). Upon splicing, a free intron lariat is excised and remains associated with its IEP, which for the *Lactococcus lactis* group II intron *Ll.LtrB* exists with a stoichiometry of 2:1 protein–RNA in a stable ribonucleoprotein (RNP) assembly (Figure 1B) (13). This protein serves a central role in facilitating the genetic mobility of the intron, by virtue of a DNA endonuclease domain (14,15). Although no experimental structure is currently available for any IEP, a homology model of the 70 kD LtrA has been derived (16) and its interactions with the intron have been mapped out *in vitro* (17). The most significant interactions between the protein and the RNA occur at domain IV, a region that is predicted to project away from the rest of the RNA core (18–20).

These findings raise fundamental questions about the role of the maturase–RNA interactions in RNP assembly and function, and the overall structural properties of group II introns. Although well-characterized on the genetic and biochemical level, biophysical and structural insights into the intact active RNP in its native form are limited. Structural studies of these large macromolecular assemblies have been challenged by the quantities of native particles typically needed for classical methods such as X-ray crystallography. While there are now experimental crystal structures of the bacterial group IIC intron RNA in different catalytic states (22,23), no structures have been reported for an intact intron RNP complex. The models available for the group IIA *Ll.LtrB* intron (20) and its IEP (16) are based on site-directed mutagenesis, chemical crosslinking and homology modeling and our only direct insight into the structure of the assembled group IIA intron have been provided by electron microscopy [EM (24)]. We recently succeeded in trapping the *Ll.LtrB* intron RNP complex in its precursor form by deleting the catalytic adenosine nucleophile in domain VI that initiates splicing. This particle was studied by sedimentation velocity, size-exclusion chromatography and cryo-EM, revealing that the intron RNP precursor (ΔA) is a large, loosely packed structure (25). The hydrodynamic properties determined for the ΔA intron contrast with those ascertained for more compactly spliced introns in a catalytically active state (+A), suggesting that a major conformational change underlies this transition.

Fundamental parameters such as size, shape and inherent flexibility of the native, active group II intron in solution have not been reported. Here, we implement a novel purification strategy to isolate homogeneous preparations of native group II intron particles from *L. lactis*. Biochemical and biophysical analyses confirm the composition 2:1 stoichiometry and monodispersity of these isolated nucleoproteins. We combine analytical ultracentrifugation, size-exclusion chromatography in-line with multi-angle light scattering (SEC-MALS), tandem mass spectrometry (LC-MS/MS) and small-angle X-ray scattering (SAXS) with contrast variation to determine the composition and structural properties of the active group II intron RNP (+A). SAXS analyses reveal an oblate ellipsoidal particle where the RNA surrounds a centrally positioned protein component. A transfer RNA (tRNA) fusion engineered into domain II of the intron provides a landmark that corroborates our interpretation of the compositional distribution of the intron in SAXS experiments. Finally, direct comparison of the SAXS-derived +A reconstruction with the previously reported ΔA EM result indicates that the precursor RNP undergoes a major conformational change adopting a more compact, ellipsoidal structure in the active form.

## MATERIALS AND METHODS

### Expression and purification of group II intron RNP particles

Details on the creation of the plasmid constructs used in this study can be found in Supplementary Methods. Transformed *L. lactis* IL1403 was grown in M17 Difico media (Becton Dickinson and Company, Franklin Lakes, NJ, USA) containing 0.5% glucose, at 30°C without aeration to an OD_600_ of 0.5–0.6 in 10 µg/ml chloramphenicol. Expression was induced with 10 ng/ml nisin for 2–3 h. The culture was then centrifuged and the pellet was washed in a buffer containing 10 mM Tris–HCl, pH 7.5, 150 mM NaCl and 1 mM ethylenediaminetetraacetic acid (EDTA). The washed cell pellet was re-suspended in 20 mM Tris–HCl, pH 8.0, 500 mM NaCl, 0.1 mM EDTA and 1 mM phenylmethanesulfonylfluoride. This cell suspension was flash-frozen in liquid N_2_ and stored at −80°C until further purification.

The purification of the active RNP using the p+A(12MS2)/LtrA (p+A) and p+A(12MS2-DIItRNA)/LtrA (p+A-DII(tRNA) constructs is similar to that reported previously for the precursor ΔA particle (25), with modifications based on MS2 tag–protein interactions to separate the precursor from the spliced intron. The MBP-MS2 binding protein (∼50 kD) used in this purification was purified as previously described (21). After the cell lysate was passed over chitin resin (New England BioLabs, Ipswitch, M.A., U.S.A.) by gravity at 4°C, the resin was washed with two column volumes of wash buffer (20 mM Tris–HCl, pH 8.0, 500 mM NaCl, 0.1 mM EDTA, 0.1% NP-40). The MBP-MS2 protein was then introduced to the resin-bound material. The resin was then washed with eight additional column volumes of wash buffer before the addition of a column volume of buffer supplemented with 40 mM dithiothreitol (DTT) to catalyze intein self-cleavage. The columns were then allowed to incubate at 4°C for 16 h to complete the reaction. The liberated group II intron RNP was eluted isocratically from the chitin resin and fractionated. To determine which fractions contained LtrA protein (and hence, intact RNP), SDS–PAGE analysis with Coomassie staining was performed. The fractions containing LtrA were pooled, concentrated and buffer-exchanged into 40 mM Tris–HCl, pH 7.5, 450 mM NaCl and 5 mM MgCl_2_ using 50 kD MWCO Amicon Ultra-15 concentrators (EMD Millipore, Darmstadt, Germany). This material was then loaded onto an isotonic 5–20% sucrose gradient cushion and centrifuged in a SW41 rotor at 4°C and 27 000 rpm for 16 h to isolate intact RNP. Additional details on the LC-MS/MS analyses of these purified particles are provided in Supplementary Methods. The composition and calculated properties of the +A components and derivatives are provided in Supplementary Table S3.

### Primer extension analysis

Primer extension was performed as described previously (26), using primer IDT1073, which is complementary to the 5′ region of the *Ll.LtrB* group II intron RNA. Primer extension products were separated by electrophoresis on denaturing 10% polyacrylamide (29:1)/8M urea/1x Tris–Borate–EDTA (TBE) gels, dried and visualized by phophorimager analysis using ImageQuant 5.2. (G.E. Healthcare Life Sciences, Piscataway, NJ, USA).

### *In vitro* reverse splicing assay

The double-stranded DNA (dsDNA) substrate was prepared as described (27). A 1 µl aliquot of purified group II intron RNP (∼100 ng/µl) was incubated with the ^32^P-labeled dsDNA (∼50 000 cpm per reaction) in a buffer containing 50 mM Tris–HCl pH 7.5, 10 mM KCl, 10 mM MgCl_2_ and 5 mM DTT (with or without 0.1% NP-40 and 0.1% Tween-20) at 37°C for various times. Reactions were terminated by phenol/chloroform extraction and ethanol precipitation. Precipitated reverse splicing products were separated by electrophoresis on denaturing 5% polyacrylamide (29:1)/8M urea/1x TBE gels, dried and visualized by phophorimager analysis using ImageQuant 5.2 (G.E. Healthcare Life Sciences).

### Size-Exclusion Chromatography in-line with Multi-angle Light Scattering

Absolute molecular weights of +A were determined by multi-angle light scattering coupled with refractive interferometric detection (Wyatt Technology Corporation, Santa Barbara, CA, USA) and a Superdex 200 10/300 GL column (G.E. Healthcare Life Sciences). The column was equilibrated in 20 mM Tris pH 7.4, 200 mM KCl and 5 mM MgCl_2,_ at room temperature. This analysis and Stokes radius (R_s_) determinations were performed as previously described (28). For determination of molecular weight of the RNP complex, a mass-averaged *dn/dc* of 0.182 ml/g was used to account for the composition of the two components of the particle.

### Analytical ultracentrifugation

Sedimentation velocity ultracentrifugation experiments were performed at 4°C with an XL-A analytical ultracentrifuge (Beckman-Coulter, IN, USA) and a TiAn60 rotor with two-channel charcoal-filled epon centerpieces and quartz windows. Samples were analyzed at concentrations of 7–14 ng/µl, as estimated by UV absorbance. Complete sedimentation velocity profiles were recorded every 30 s at ABS_260_ nm for 50–200 boundaries at 28 000 rpm. Data were fit using the Lamm equation as implemented in the program SEDFIT (29). After optimizing meniscus position and fitting limits, the sedimentation coefficient (*S*) and best-fit frictional ratio (*f/f_0_*) was determined by iterative least squares analysis. Analyses were carried out in buffer composed of 20 mM Tris pH 7.4, 200 mM KCl and 5 mM MgCl_2_. The solvent density (*ρ* = 1.0086 g/ml) and viscosity (*η* = 0.01007 poise) were derived from chemical composition by the program SEDNTERP (30). A partial-specific volume (ν) value of 0.630 cm^3^/g was used for the +A particle, as previously estimated (25). In these experimental conditions, the pressure encountered by the particles range from ∼25 to 50 bar between the middle and bottom of the cell, as calculated by SEDFIT. Theoretical data for simulation of particles of given mass but different shapes were generated using the SEDFIT program.

### SAXS data collection

X-ray scattering data were measured at the F2 and G1 beam lines at the Cornell University High-Energy Synchrotron Source (CHESS, Ithaca, NY, USA), the BIOCAT beam line at the Advanced Photon Source (APS, Argonne, IL, USA) and beam line X9 at the National Synchrotron Light Source (NSLS, Upton, NY, USA). Specific details of the experimental set-up and procedures specific to each location are provided in the Supplementary Methods. In all cases, the forward scattering from the samples studied was recorded on a CCD or Pilatus (Dectris Ltd., Baden, Switzerland) detector and circularly averaged to yield one-dimensional intensity profiles as a function of *q* (*q* = 4πsinθ/λ, where 2θ is the scattering angle in units of Å^-1^). For final data collection, samples were exchanged into 20 mM Tris pH 7.4, 200 mM KCl and 5 mM MgCl_2_ at 4°C using 10 kD MWCO Amicon Ultra-15 concentrators (EMD Millipore) and centrifuged through 0.45 µm Whatman spin filters (Fisher Scientific, Pittsburgh, PA, USA) before 0.5–30 s exposures were recorded at 4°C. Scattering from a matching buffer solution was subtracted from the data and corrected for the incident intensity of X-rays. Beam attenuation was employed and replicate exposures were examined carefully for evidence of radiation damage by Guinier and Kratky plot analysis. Silver behenate powder was used to calibrate the sample-to-detector distance and beam center. Additional details on SAXS data analysis and shape reconstruction are available in Supplementary Methods.

## RESULTS

### Purification of native LtrB group II intron RNP

**Figure 2.**
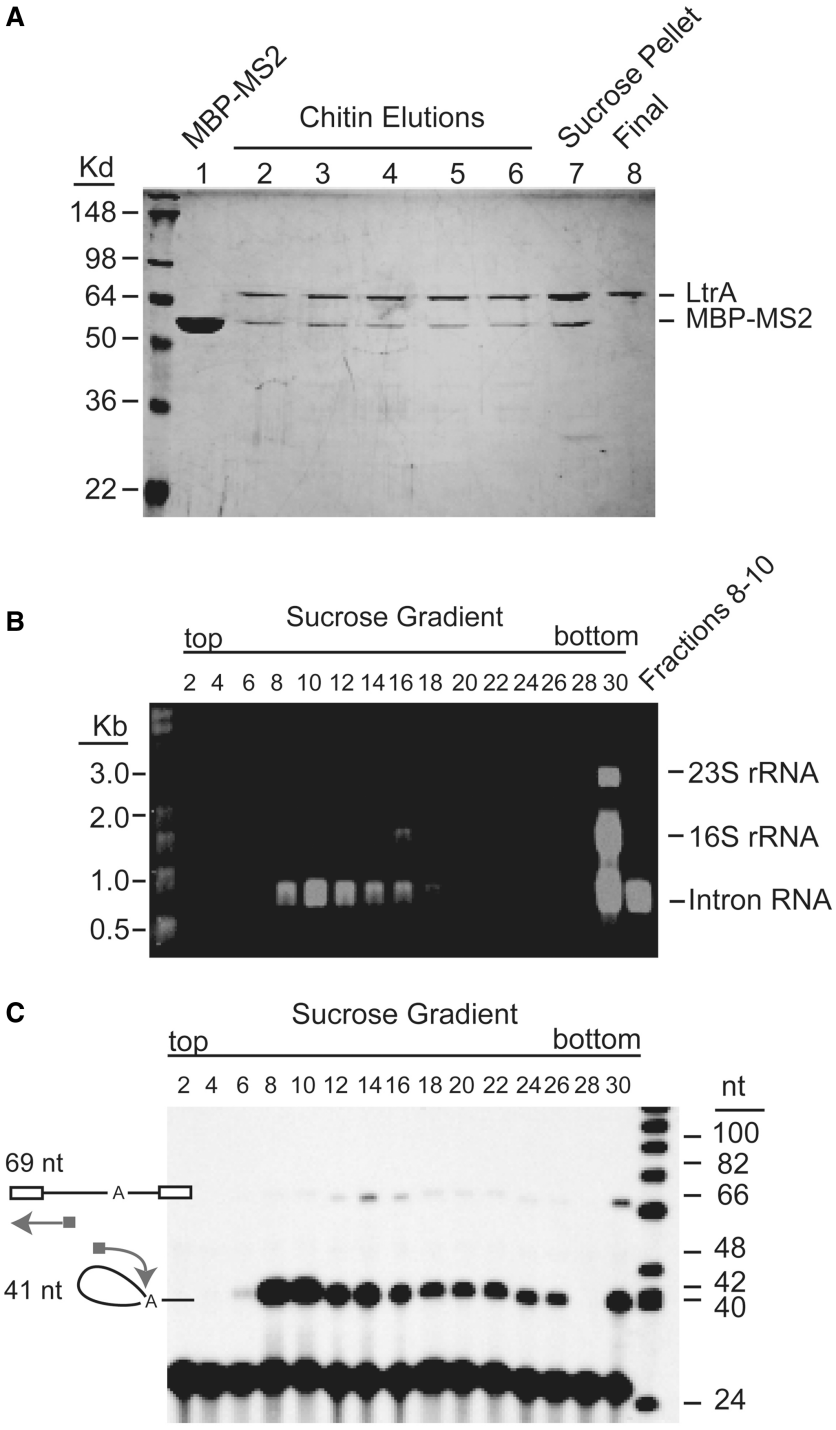
Purification of the active intron RNP. (**A**) SDS–PAGE analysis. Purified intron RNP contains a single protein band at an apparent mass of ∼70 kD corresponding to the IEP, LtrA (lane 8) (27). Lane 1: purified MS2–MBP fusion protein, lanes 2–6: chitin elution fractions; lane 7: re-suspension of the pellet from sucrose cushion centrifugation; lane 8: purified RNP. (**B**) RNA analysis of the purified intron RNP. The RNA content of alternating sucrose gradient fractions was analyzed on a 1.2% agarose gel containing 2.2 M formaldehyde. Purified intron RNP in fractions 8–10 was pooled and concentrated. The RNA content of the pooled chitin elution and the re-suspension of the sucrose cushion pellet were also analyzed, with a band of ∼902 nt corresponding to the size of the LtrB intron lariat (data not shown). (**C**) Primer extension assay. A primer was designed to target the 5′-end of the LtrB intron. A 69-nt primer extension product was generated from LtrB intron precursor and a 41-nt primer extension product was generated from LtrB intron lariat. Pure lariats (in the absence of the precursor) were isolated in the collected peak fractions 8–10.

We designed an affinity purification scheme to specifically isolate the LtrB group II intron RNP in its active state (+A) from its native *L. lactis* host. The overall strategy is modified from that employed previously for purification of the precursor particle [ΔA,(25)], by employing MS2 tag–protein interactions to purify spliced intron away from the precursor. In this scheme, the intron and the IEP (LtrA) components are expressed under the control of independent nisin A promoters (Figure 1C), allowing for formation of the RNP in *L. lactis*. To facilitate purification, a C-terminal chitin binding domain (CBD) was fused to LtrA via an intein. A truncated version of the intron RNA missing 1590 of the 1797-nt LtrA open reading frame (ΔORF) was expressed upstream of the LtrA-intein-CBD cassette. Additionally, the fusion transcript was tagged with a 12xMS2 tag (31) on its 3′ exon (Figure 1C). Upon activation with thiol reagents, the intein cleaves at its N-terminus, releasing LtrA and its tightly associated *Ll.LtrB* group II intron from the chitin resin. *In vivo* expression of this active construct yields a mixture of RNPs: both unprocessed precursor and spliced lariats (Figure 1C). The addition of the 12xMS2 tag (∼1000 nt) to the exon in the presence of MS2-maltose-binding protein (MBP) provides a strategic size differential between the precursor and the excised lariat for separation using a sucrose gradient. SDS–PAGE analysis of the protein content of the RNP captured on the chitin resin (Figure 2A, lanes 2–6) shows LtrA, the fusion MBP–MS2 (Figure 2A, lane 1) and minor contaminants. The final RNP product (Figure 2A, lane 8) showed a single band for the 70-kD LtrA protein after fractionation on the sucrose gradient.

The intron RNA lariats (902 nt) co-purify with 16S ribosomal RNA from the 30S ribosome (Figure 2B) as was the case in the purification of the ΔA precursor particle (25). Fractionation by sucrose gradient allows for isolation of +A particle both alone (Figure 2B, fractions 8–10) and in complex with this 30S ribosomal species (Figure 2B, fractions 12–30). The purity and identity of the protein and nucleic acid components of the +A preparations were confirmed by western blot analysis for the LtrA component and northern blot analysis of the intron component (data not shown). Tandem mass spectrometry analysis confirms the presence of intact LtrA protein in RNP preparations (Supplementary Figure S1). The high-affinity association between the intron RNPs and ribosomes protects the intron from ribonuclease degradation (32).

To confirm that the purified RNPs did not contain a mixture with precursor particles we conducted a primer extension termination assay on all the fractions collected from the sucrose gradient (Figure 2C). Here, we separated cDNA products of the RNAs by denaturing PAGE and determined that the main peak fractions collected during purification (Figure 2B, fractions 8–10) contained an abundance of cDNA corresponding to excised intron (41 nt) and were virtually free of the cDNAs corresponding to precursor (69 nt) (Figure 2C). These analyses confirmed that the purification scheme employed was successful in isolating homogeneous particles of the active RNP.

### Functional assays confirm that the RNP is an active particle

**Figure 3.**
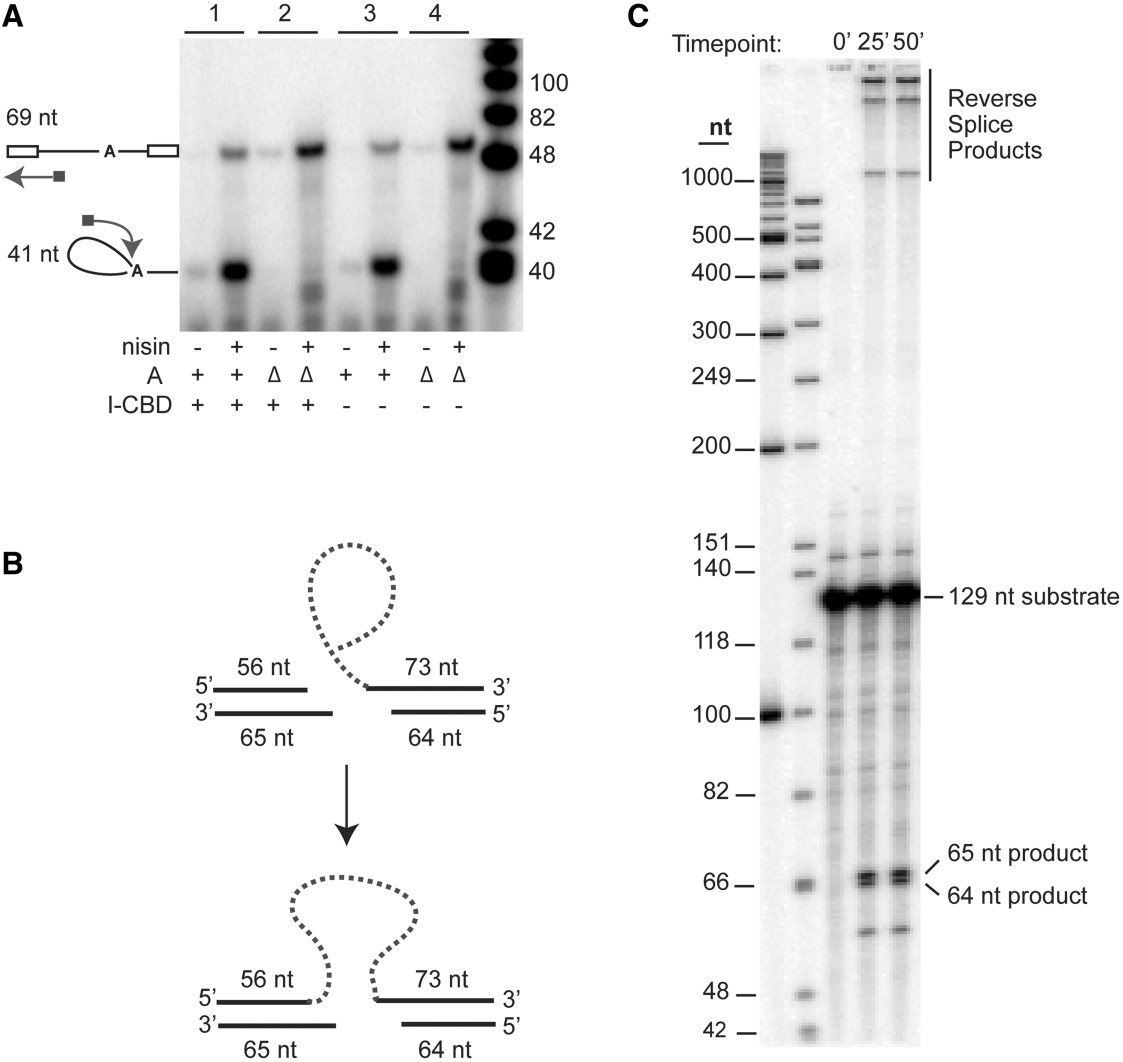
Activity assays. (**A**) Primer extension assay. Assays were performed as in Figure 2C. Samples in each pair of lanes were uninduced (−) or induced (+) with nisin. The splicing-proficient RNPs (+A, samples 1 and 3) were compared with splicing-defective RNPs (ΔA, samples 2 and 4). Fusion constructs containing the intein and CBD (I-CBD) are in samples 1 and 2. (**B**) Schematic description of the *in vitro* reverse splicing assay. (**C**) Reverse splicing assay. Purified intron RNP was incubated with internally ^32^P-labeled dsDNA substrate containing a 129-bp homing site, at 37°C for varying times, quenched using 1:1 phenol–chloroform and separated on a 5% denaturing PAGE gel. The radiolabeled 129-nt substrate and the 65- and 64-nt product bands are indicated. The highest molecular weight bands (>1000 nt) are reverse splicing products of the lariats on the sense strand of the dsDNA.

We next confirmed the activity of the product of our expression constructs both *in vivo*, before purification, and *in vitro*, after purification. Using the primer-extension assay in Figure 2C, we assayed RNA splicing from actively growing cells and showed that the splicing efficiency of the construct with an LtrA fusion to the intein and CBD was equivalent to that with unfused LtrA (Figure 3A). The precursor–product ratios were similar in the two cases (*cf*. lanes 1 and 3), whereas the non-splicing ΔA constructs showed no products (lanes 2 and 4). Likewise, in a genetic assay that measures retrohoming into a plasmid recipient, the group II intron was inherited at ∼60% of the wild-type from a donor containing the LtrA fusion, despite the potential for steric hindrance by the fusion protein (data not shown).

We next analyzed the activity of the purified particles, by testing the ability of the intron RNA in the native +A RNP to reverse splice into a 129-bp internally labeled DNA substrate, which contains the exon1–exon2 junction of the *ltrB* gene (Figure 3B and C). The RNP particles collected from each fraction of the sucrose gradient were analyzed for cleavage products by denaturing PAGE analysis (Figure 3C). Endonuclease activity was evidenced by the cleavage of the antisense strand of the dsDNA substrate at position +9, yielding two cleavage products of 64 and 65 nt. Additionally, the slower migrating bands at the top of the gel reflect complete reverse splicing of the lariats on the sense strand of the dsDNA.

### Monodispersity, stoichiometry and hydrodynamic properties of +A preparations

**Figure 4.**
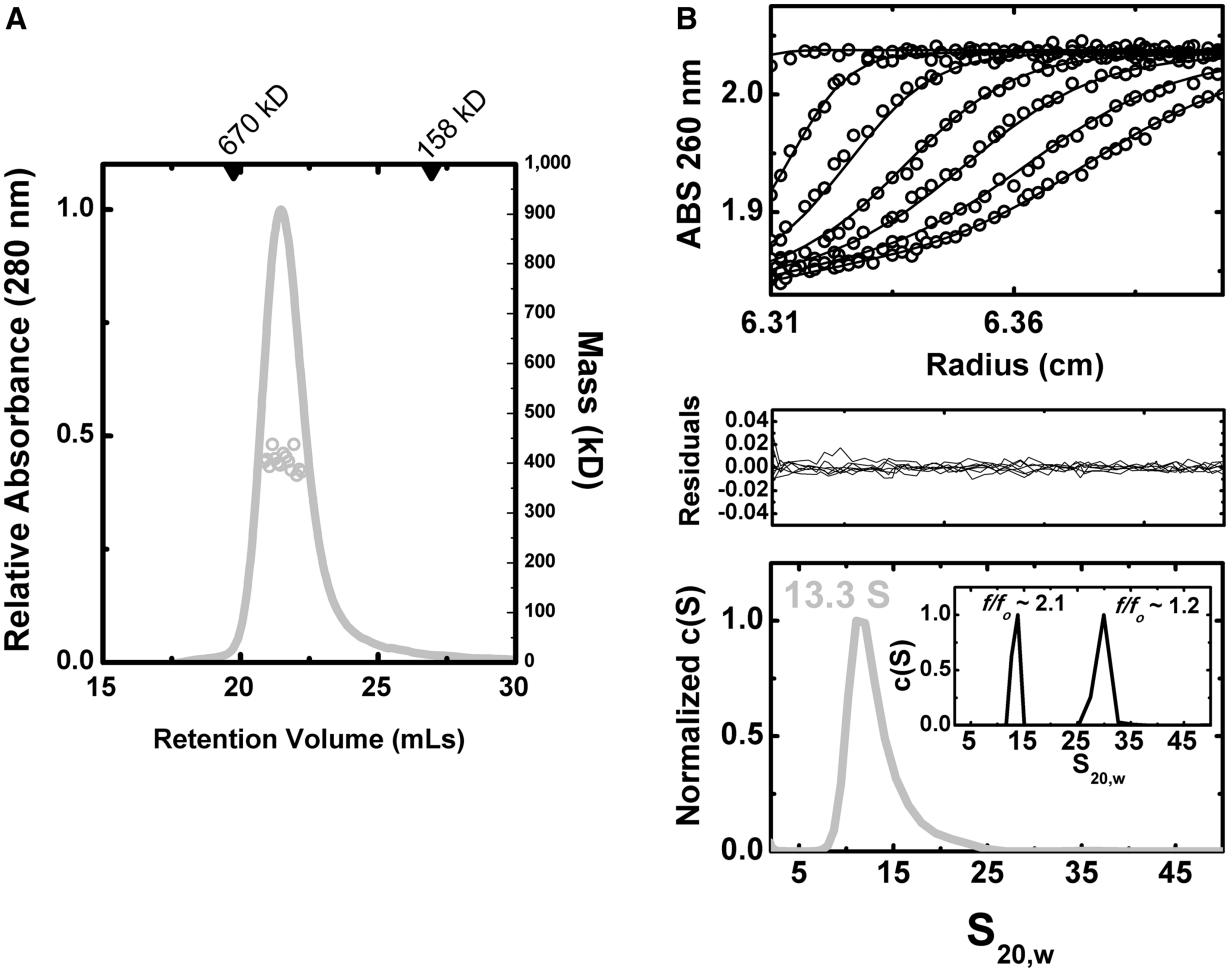
Biophysical analysis of RNP particles. (**A**) Representative SEC-MALS analysis. Shown as a gray line is the absorbance profile of sample, as a function of elution time from a Superdex-200 10/300 column at room temperature (left axis). Corresponding gray circles denote molecular masses determined by in-line light scattering (right axis). Across the half-peak, the determined *M*_w_ was 404 kD ±0.6% using a mass-averaged *dn/dc*, consistent with a predicted molecular weight of 432 kD. (**B**) Sedimentation velocity analytical ultracentrifugation. c(S) distributions (lowest panel) were derived from the fitting of the Lamm equation to the experimental data collected, as implemented in the program SEDFIT (29). Shown in the inset of the bottom panel are simulated c(S) distributions for a particle of identical mass and composition to +A but with different frictional coefficients, corresponding to spherical (*f/f_o_* ∼1.2) and elongated (*f/f_o_* ∼2.1) forms. In the uppermost panel, fits of the experimental data (black circles) to the Lamm equation are shown as black lines; in middle panel the residuals from this fitting are shown. Measurements were performed at relatively low molar concentrations (∼10 ng/µl) and indicated a single species. Consistent with previous measurements, the +A particle showed a single peak with an *S*_20,w_ value of 13.3 and a frictional coefficient (*f/f_o_*) of 2.1.

We first addressed the concentration-dependent behavior and self-association properties of the +A particle. As determined by SEC-MALS (Figure 4A), the purified +A particles are monodisperse. The +A particle elutes as a single species with an apparent mass of 483 kD against a standard curve derived from globular protein standards. Both this apparent mass and the determined mass (*M*_w_) by MALS of 404 kD ±0.6% across the peak half-height are consistent with a calculated theoretical mass of 432 kD for a complex comprising a 140 kD LtrA dimer bound to a 902 nt RNA (Supplementary Table S3). These results corroborate our previous determinations of mass for these preparations (25) and prior reports of a 2:1 protein–RNA stoichiometry (13). As determined by refractive index, the eluted material was at very low molar concentration, consistent with previous reports of low nanomolar to picomolar binding affinities between the protein and RNA components *in vitro* (13,18,33).

These measurements were complemented by determination of the hydrodynamic properties of the purified +A RNP using sedimentation velocity analytical ultracentrifugation (Figure 4B). Measurements performed at 7–14 ng/µl concentration indicated single species, consistent with previous analyses of +A and ΔA particles alone in comparable sample conditions (25). The absorbance data collected for the +A RNP was readily fit to the Lamm equation across all the time boundaries collected, yielding a single peak in the *c*(S) distribution with an S_20,w_ value of 13.3 and a frictional coefficient (*f/f_o_*) of 2.1. This latter value indicates a non-spherical particle, with calculated axial ratios (*a/b*) of 18–20 for oblate or prolate ellipsoid models, respectively. For reference, a spherical particle (*f/f_o_* ∼1.2) of this composition and mass would appear as a ∼30S species (Figure 4B). Using the experimentally determined parameters from sedimentation velocity and a mass-averaged partial specific volume, an estimated mass of 453 kD was derived, again in line with the predicted mass of a 2:1 complex.

### Shape and volumetric properties of native group II intron particles determined by SAXS

**Figure 5.**
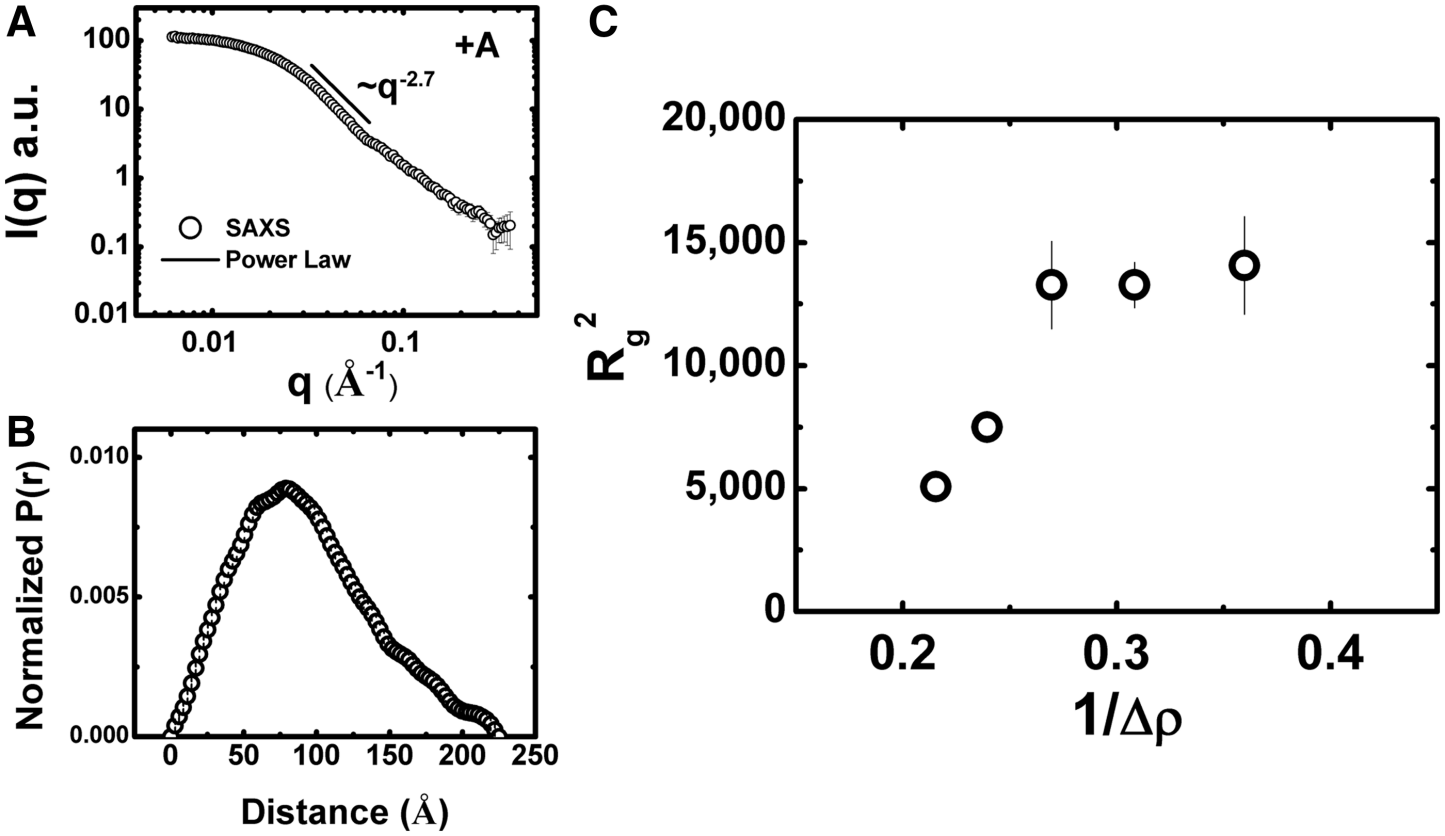
SAXS Analysis of +A RNPs. (**A**) Representative SAXS data from +A (black open circles). The recorded intensity is shown as a function of *q* (*q* = 4πsinθ/λ, where 2θ is the scattering angle). Parameters derived from this analysis are summarized in Table 1. Error bars represent plus and minus the combined standard uncertainty of the data collection. Depicted with a black line is slope of the decay in the middle *q* regime. This region decays with ∼*q*^2.7^, indicative of an oblate ellipsoid. (**B**) Pr analysis. This analysis is derived from the inverse Fourier transform of the primary scattering data, providing a shape distribution function depicting the distribution of the interatomic vectors in the particle. (**C**) Contrast variation analysis. Stuhrmann plot of the *R*_g_ values determined for the +A RNP as a function of the reciprocal of contrast (as conferred by increasing amounts of sucrose). The experimental data points are shown as open circles. The error bars represent the statistical uncertainty associated with measurement.

We extended our analysis to include SAXS, a technique well-suited to characterize large native RNP particles available in modest quantities. The technique has found extensive application to the study of the ribosome (34,35). Although inherently low in spatial resolution, the technique provides the gross structural and compositional properties of particles in solution in a model-independent fashion. Due to the limited microgram quantities of purified +A particles available, several experimental considerations were made and optimized to facilitate this study (Supplementary Text). Using synchrotron radiation, the +A particle had a radius of gyration (*R*_g_) of ∼73 Å and a maximum dimension (*D*_max_) of ∼235 Å (Figure 5A and Table 1), with structural parameters derived from classical Guinier analysis and the indirect Fourier transform yielding comparable results. These SAXS-derived parameters are comparable to those previously determined for 30S ribosomes. For example, the *R*_g_ and *D*_max_ of an oblate ellipsoidal 489 kD 30S ribosomal particle from *Sulfolobus solfataricus* (∼59% nucleic acid) are ∼69 Å and 230 Å, respectively, based on SAXS analysis (36). In contrast, the SAXS-derived *R*_g_ and *D*_max_ of the smaller (∼200 kD) mononucleosome particle (an oblate ellipsoid) that is ∼50% nucleic acid is ∼44 Å and ∼129 Å, respectively (37). Hence, the structural parameters determined by SAXS for the +A RNP are consistent with a nucleoprotein complex of its mass and composition. As determined by Kratky and Porod-Debye analyses (Supplementary Figure S3), some inherent flexibility in the +A particle is indicated.

**Table 1.** Parameters derived from SAXS

		Guinier			GNOM			
Sample	Conc.^a^ (ng/µl)	q*R*_g_	*R*_g_ (Å)	I(0) (a.u.)	q^b^	*R*_g_ (Å)	I(0)	*D*_max_ (Å)
APS BioCAT								
+A Group II Intron	100	0.98–1.3	73.5 ± 0.8	324.0 ± 3.0	0.006–0.37	74.9	326.2	250
	50	0.47–1.3	75.3 ± 0.9	139.0 ± 1.0	0.006–0.37	72.7	133.0	225
	40	0.45–1.3	73.0 ± 1.0	120.2 ± 1.0	0.006–0.31	72.4	116.0	225
	*Inf. dil.*	0.57–1.2	73.3 ± 0.9			73.3		235
NSLS X9
+A Group II Intron (DII-tRNA)	100	1.0–1.4	87.3 ± 1.2	131.7 ± 2.1	0.010–0.25	95.1	125.0	284
CHESS F2^c^
+A Group II Intron								
0% Sucrose	40	0.71–1.3	71.2 ± 4.4	0.53 ± 0.03	0.0097–0.15	70.7	0.52	235
16.5% Sucrose	60	0.89–1.7	86.6 ± 3.9	0.70 ± 0.04	0.0097–0.15	83.8	0.64	235
32.5% Sucrose	50	1.09–1.9	105.8 ± 8.3	0.52 ± 0.06	0.0097–0.10	92.9	0.42	235
48.8% Sucrose	100	1.19–2.1	115.2 ± 5.6	0.87 ± 0.07	-n.d.-	-n.d.-	-n.d.-	
65% Sucrose	50	1.22–2.2	118.6 ± 11.8	0.41 ± 0.07	-n.d.-	-n.d.-	-n.d.-	

^a^Where q = 4πsinθ/λ, where 2θ is the scattering angle, in units of Å^-1^.

^b^As determined by U.V. absorbance.

^c^I(0) in units of (cm^-1^)

The asymmetric shape distribution (Pr) profile for the +A RNP is consistent with an ellipsoidal shape (Figure 5B). Consistent with this interpretation, we found that the experimental data at lower scattering angles was well described with an ellipsoid form factor with dimensions of ∼218 × ∼218 × ∼34 Å and a hydrated volume of ∼855 000 Å^3^ (*χ* = 1.93, data not shown), indicating an oblate ellipsoidal shape. This oblate character is further supported by the decay of scattering intensity observed in the experimental profile. For oblate particles, intensity follows approximate q^-2^ dependence, whereas for prolate ellipsoids, the decay is better described by a q^-1^ relationship (38). In the +A RNP data, the decay varies with ∼1/q^2.7^ (Figure 5A). Thus, SAXS and SV analyses both indicate that the +A particle is an ellipsoid of oblate character.

### Contrast variation analysis

The SAXS analyses described above assume that the particle is homogeneous. However, the +A RNP is a composite particle consisting of both protein and RNA, where the RNA contributes more scattering power per unit mass. To evaluate the relative contributions of protein versus RNA to the overall shape of the +A particle, we combined contrast variation with SAXS analysis. Contrast variation using small-angle neutron scattering (SANS) is typically performed by selective deuteration, or more simply, by modification of the scattering density of the solvent using D_2_O. The limited quantity of +A RNP particles available precluded the application of SANS, in our study, but contrast variation can be achieved in X-ray scattering by addition of electron-rich additives such as glycerol, salts or sucrose. As the RNP particles in this study have proven to be stable in the high percentage sucrose gradients used in their preparation, we performed contrast variation with X-ray scattering using sucrose.

Theoretical calculations indicated that increasing sucrose concentrations would be expected to amplify the contribution of the RNA component and diminish the contribution of the protein component to overall *R*_g_ (which represents the root mean square distance of all of the interatomic vectors relative to the center of mass). The trend in *R*_g_ as a function of contrast provides direct insight into the spatial arrangement of the components within the complex. We hypothesized that if LtrA is positioned on the outside of the complex, away from the center of mass, we would expect *R*_g_ to decrease with increasing contrast. Alternatively, if LtrA is located near the center of mass, we would expect *R*_g_ to increase, as the protein’s contribution to the smallest interatomic vectors within the total particle would be diminished.

The data obtained by this approach are shown in Figure 5C, Table 1 and Supplementary Figure S2. With increasing sucrose concentrations, *R*_g_ increased dramatically, with a plateau ∼115 Å, indicating that the lower-density protein component of the RNP lies near the center of mass of the particle. The contrast variation results also indicate that the RNA is distributed over a large volume, possibly a consequence of wrapping around a central protein core. Indeed, the apparent *R*_g_ provided by the RNA component is surprisingly large, given that Flory’s law predicts an *R*_g_ of 53 Å for a compactly folded RNA of this composition (39).

The variation in observed *R*_g_ with contrast (Δρ) shown in Figure 5C can be described by the Stuhrmann equation (40):

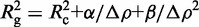

Where *R*_c_ is the *R*_g_ at infinite contrast, the constant α describes the inhomogeneity of the particle and β the displacement of the center of the scattering length density from the center of mass. While the limited range of contrast data obtained precludes rigorous quantitative analysis by this relationship (including a determination of the *R*_g_ at infinite contrast), the trends in the data clearly indicate a positive value for the α term. This result is similar to that observed for the nucleosome, where the more electron dense component is located near the periphery of the particle (41). A non-zero value for the β term, indicated by the nonlinear relationship between *R*_g_ and contrast, suggests an asymmetric arrangement of protein and nucleic acid components relative to the center of mass of the +A particle.

### Introduction of a tRNA landmark into domain II of the +A particle

**Figure 6.**
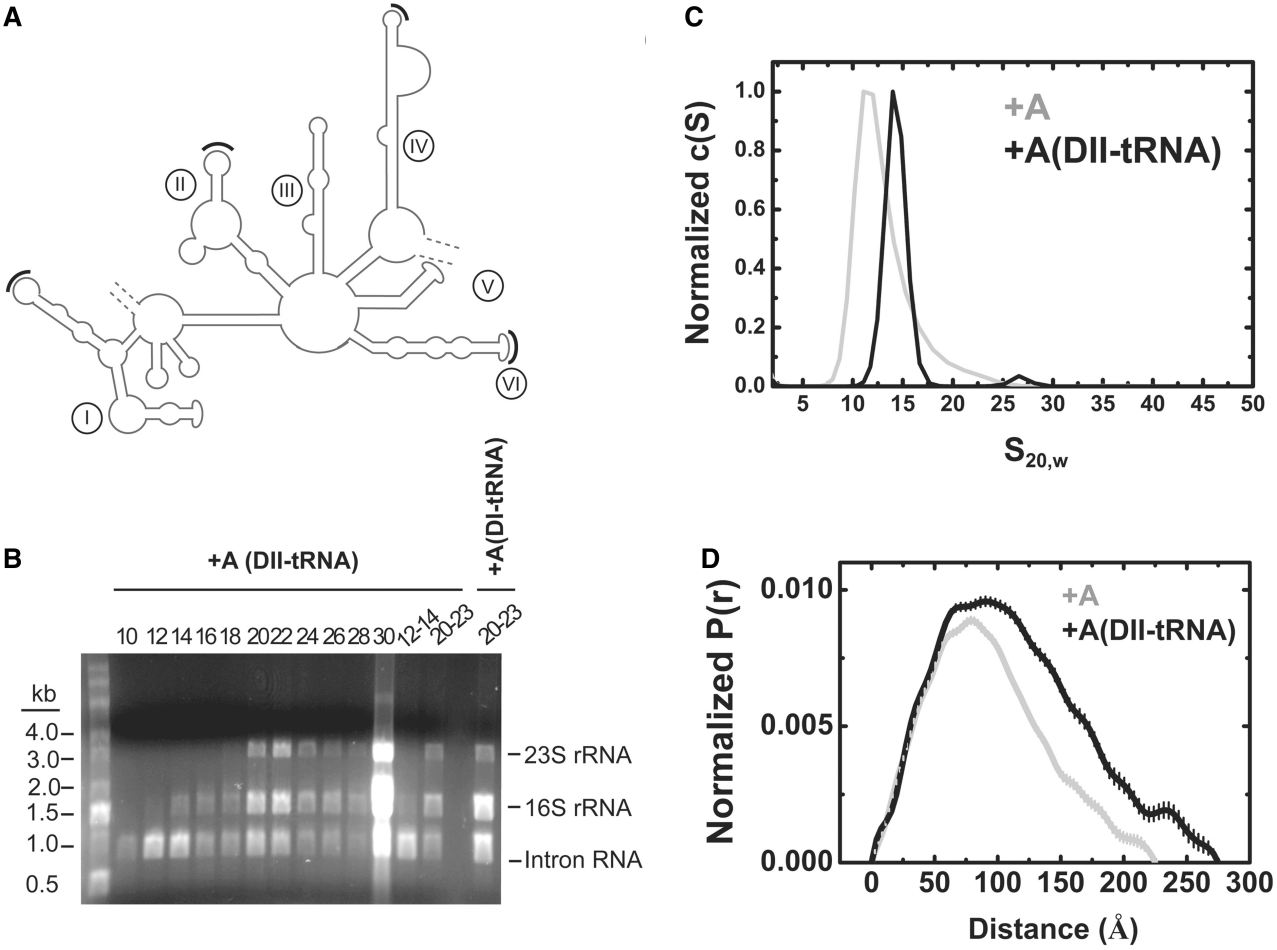
Properties of a tRNA-containing group II intron. (**A**) Secondary structure map of the Ll.LtrB intron with black arcs showing sites of tRNA insertion in domains I, II, IV and VI. In all four cases, a Bglll site AGATCT was created in the terminal loop from the following sequences AGACAA (I), AGCACU (II), AAACCU (IV) and AGUAAU (VI) for integration of the tRNA sequences. (**B**) RNA analysis of purified +A-DII (tRNA) intron. The RNA content of alternating sucrose gradient fractions are shown separated on a 1.2% agarose gel containing 2.2 M formaldehyde as in Figure 2B. Pooled fractions 12–14 contain pure intron, whereas pooled fractions 20–23 contain rRNAs as well. In contrast the +A-DI (tRNA) construct on the right yielded no pure intron (blank lane, fractions 12–14) but the pooled fractions 20–23 show intron with rRNA. (**C**) Sedimentation velocity analysis. c(S) distributions are shown that compare the properties of the +A RNP (red) to that of the DII(tRNA) insertion construct (blue). (**D**) Pr analysis. Pr analysis derived from SAXS analysis, comparing the spatial properties of the +A (red) and +A-DII(tRNA) (blue) constructs.

To further probe the structural arrangement of the +A particle, we engineered tRNAs into selected locations into domains I, II, IV and VI of the intron (Figure 6A). All four of the ΔORF-12MS2 tRNA derivatives had splicing activity (Supplementary Figure S4A), but only the domain II construct [p+A-DII(tRNA)] was well behaved during purification (Figure 6B). The free intron peaked in fractions 12–14 of the sucrose gradient in a similar range to its parental +A counterpart. In both DNA-binding assays (Supplementary Figure S4B) and reverse splicing assays (Supplementary Figure S4C) the +A-DII(tRNA) construct had similar activity to its +A counterpart.

At 20°C, the +A-DII(tRNA) particles were not sufficiently monodisperse for hydrodynamic and scattering analyses (Supplementary Figure S5A). However, at 4°C sedimentation velocity analysis indicated comparable behavior to the native +A particle during the initial stages of centrifugation (Figure 6C). As the centrifugation experiment continued, the +A-DII(tRNA) particles were susceptible to pressure-induced dissociation (Supplementary Figure S5B). This perturbation of the innate stability of the +A particle by the cell pressures associated with high rotor speeds is consistent with the view that domain II lies proximal to the protein–RNA interface (42,43) and possible weakening of the interaction by the tRNA insertion.

Accordingly, our analyses of the RNP particles in this study were performed at 4°C. Based on SAXS analysis, the +A-DII(tRNA) particle features spatial properties significantly larger than those observed for the +A particle alone, as evidenced by *R*_g_ and *D*_max_ (90 Å and 270 Å, Table 1). Differences in the Pr distributions occur at the largest interatomic vectors, consistent with a distal placement of the tRNA component relative to the center of mass of the complex (Figure 6D).

### Reconstruction of the low resolution structure of the +A group II intron from solution scattering

To model the relative positions of the protein and RNA components of the +A particle, modeling was performed using a multi-phase bead approach to shape restoration (44). With this method, two phases of different scattering length densities were derived using the 0, 16 and 32% sucrose experimental data and incorporating calculated scattering length densities. Higher angle scattering data, in general contain contributions of internal particle structure. As this approach does not attempt to model this internal structure and assume more uniform densities for the two phases, only the highest signal-to-noise data in the low *q* regime of data were employed (45).

**Figure 7.**
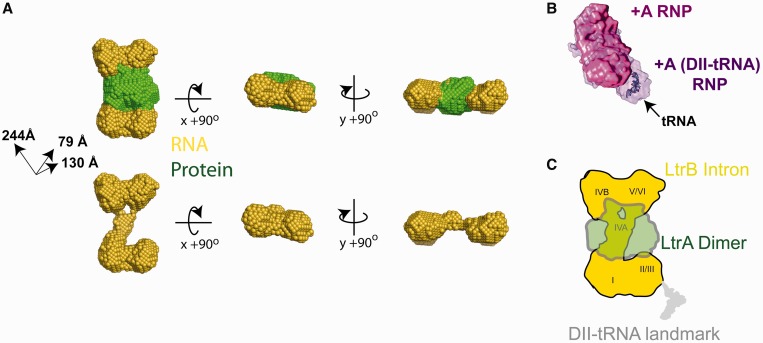
Shape reconstruction of the +A RNP particle. (**A**) The final averaged and filtered two phase shape was calculated by MONSA, shown in orthogonal views. The upper series shows the reconstruction with both RNA (yellow spheres) and protein (green spheres) phases (upper series) and with the RNA phase only (lower series). The final overall particle has dimensions of ∼244 × ∼140 × ∼80 Å. The final NSD figure for the averaged and filtered envelope is 0.77 ± 0.16. (**B**) Manual superposition of SAXS reconstructions for +A (magenta) and +A-DII(tRNA) (purple), revealing the different spatial feature assigned to the tRNA insertion. The available crystallographic structure of phenylalanine-tRNA [2TRA (46), blue] is manually docked into this lobe of difference density. The +A-DII(tRNA) variant is a prolate ellipsoid with comparable dimensions of 177 × 181 × 260 Å. Galleries of individual reconstructions for both particles are provided in Supplementary Figure S5. (**C**) Speculative model for the quaternary arrangement of the +A RNP. Roman numerals denote the speculative placement of the structural domains of the RNA component (yellow) based on the tRNA insertion data, connectivity and percentage volumes estimated for each domain based on sequence and the requirement for LtrA dimer binding to domain IV.

The results for mutiphase reconstruction are shown in Figure 7 and Supplementary Figure S2. Ten independent calculations yielded similar results, with *χ* values ranging from 1.18 to 1.39 for each of the three scattering profiles used. Reconstructions of the +A particle featured normalized spatial discrepancy (NSD) values ranging from 0.69 to 0.82, which for an anisotropic particle indicates a reasonably stable solution. These 10 calculations were combined to yield the final averaged and filtered reconstruction (Supplementary Figure S2).

The +A RNP bead model is an oblate ellipsoid with dimensions of 244 × 244 × 79 Å, with calculated sedimentation coefficient and Stokes radius values consistent with those determined experimentally (Table 2). The protein component of this two-phase model is seen to occupy a position at the center of mass, in line with contrast variation results, whereas the RNA phase is seen to assume an extended ‘bow-tie’-like arrangement, in which two larger lobes of RNA density are separated by long extension. This overall arrangement is reminiscent of a similarly described ‘chain-link’ class of shapes observed by cryo-EM with the ΔA particle (25). The protein phase of +A is seen to lie immediately adjacent to a single long extension of the RNA phase which interconnects the remaining two larger lobes. By virtue of its high-affinity primary interaction with LtrA (13,18,33), it can be inferred that this region of the RNA corresponds to domain IV.

**Table 2.** Comparison of properties of +A and ΔA experimental shapes with their experimental and calculated properties

Model	Experimental model					Sedimentation velocity^b^		SEC^c^	Calculated		
	*R*_s_(Å)	*R*_g_(Å)	*D*_max_(Å)	Volume(Å^3^)	*S*_T,b_(*f/f_o_)*	*S*_20,w_ (*f/f_o_)*	MW (kD)	*R*_s_ (Å)	Volume(Å^3^)	MW (kD)	*ʋ*_bar_ (cm^3^/g) ^g^
**+A (SAXS)****^a^**	89.5	77.5	275	940 300	15.6	13.3 (2.1)	453	77	450 339	432	0.63
**ΔA (EM) (24)**	101.0	83.9	267	2 974 000	14.4	19.7 (1.6)	473	116	475 124	458	0.62

^a^This work. The calculation of hydrodynamic properties from shape reconstructions are described in Supplementary Methods.

^b^Measurement performed at 4°C.

^c^Measurement performed at room temperature.

While SAXS measurements without contrast variation lack the ability to discern protein from nucleic acid, it is expected that relative comparisons remain informative and are generally representative of gross structural properties. To ascertain the position of the tRNA insert in the +A-DII(tRNA), solution shapes for both +A and the DII(tRNA) variant particles were each reconstructed to the same nominal scattering angle. Thirty independent reconstructions resulted in very similar shapes (Supplementary Figure S6). Although both particles have generally consistent overall properties, the +A-DII(tRNA) features an additional pronounced lobe on the distal end of the particle, extending its effective length (Figure 7B). Manual superposition of these two envelopes reveals a distinct lobe of density corresponding to the tRNA insert and hence, the vicinity of domain II within the intron component. The volume of this difference density is consistent with the known structure of tRNA when manually docked (Figure 7B). The general concordance of the shapes determined and the agreement of the difference volume with available structures of tRNA also suggests that the insertion does not fundamentally alter the native gross properties of the +A RNP structure.

### Reconciliation of the low resolution molecular envelope of the +A group II intron with available structural models

**Figure 8.**
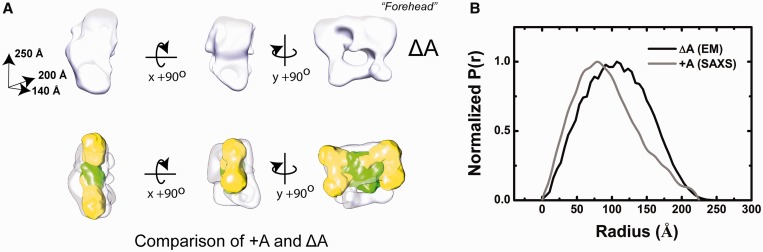
Comparison of the +A and ΔA experimental models. (**A**) The ΔA cryo-EM reconstruction. The model [white, (25)] is shown in orthogonal views. (**B**) Manual docking of the SAXS-derived +A particle (yellow phase for RNA and green phase for protein) into the ΔA shape, revealing spatial differences in RNP structure that coincide with the transition from the precursor to final states. (**C**) Shape distribution function analysis, comparing the interatomic vector distribution of the +A particle (SAXS, magenta) and ΔA shapes (cryo-EM, gray).

Our results provide the opportunity for direct comparison of the +A particle with the spatial features of the ΔA precursor particle previously derived by electron microscopy (25). ΔA is a 250 × 200 × 140 Å particle with an apparent porosity we previously hypothesized to account for its unusually large volume-to-mass ratio (24,25) (Figure 8A). The bulk of the volume of the particle is defined by a main lobe of elongated volume referred to as the ‘forehead’ feature. Similarities with the structure determined for the +A particle are readily apparent and manual docking of the +A SAXS reconstruction into the main lobe of the ΔA EM reconstruction further underscores these similarities (Figure 8A). The spatial properties of the +A shape correlate well with the main ‘forehead’ lobe of the ΔA particle, possibly defining a core body preserved in the transition from precursor to spliced state. Taken together, the differences observed between the ΔA and +A shapes indicates an asymmetric and dramatic conformational change that compacts the intron particle from an extended state to a more compact form. These observations are further corroborated by comparison of the calculated Pr function for the cryo-EM ΔA shape with that determined by SAXS for the +A particle (Figure 8B and Table 2).

## DISCUSSION

In this study, we have defined biochemical, biophysical and structural properties of the native (+A) group II intron from *L. lactis*, poised for attack of a DNA target. We reconciled these properties with those already determined for the intron in its precursor state (24,25). SAXS is a long-established technique that has been valuable in understanding the properties of native RNPs, including prokaryotic ribosomes (34,35) and other nucleoprotein assemblies like the nucleosome (37,47,48). The combination of scattering methods with the biochemical and biophysical experiments presented in this report has led to an understanding of the properties of a group II intron RNP as it exists in its native state in solution The experiments have also allowed us to propose a coarse-grain quaternary model for the RNP complex to guide future studies (Figure 7C).

Although purified group II introns RNPs formed in native conditions are highly active, our experimental approach only yields ∼1.5 µg RNP/l of culture. These quantities have proved sufficient for study by methods such as electron microscopy (25), mass spectrometry, analytical centrifugation, light scattering and SAXS. Our work with these particles confirms the composition, stoichiometry, and high-affinity interaction between the two components of the +A particle previously determined by *in vitro* analyses (13). This work is a critical first step towards the long term goal of determination of three-dimensional structures of these RNPs and will guide forthcoming cryo-EM reconstructions on the +A particle.

In contrast to previous studies of the particle in its precursor state (ΔA), a relatively more compact group II intron RNP is observed here, with an atomic volume that reconciles well with its composition and stoichiometry. The contrast that is drawn with the experimental structures of its precursor state (ΔA) provides important insight into the dramatic nature of the structural changes that occur upon formation of the native active state. Lacking in the +A shape reconstruction are the porous features that mark the experimental shape of the ΔA particle as well as similar cavities observed in cryo-EM studies of the spliceosome (49,50). This is to be expected, as the shape reconstruction algorithms utilized in this study generally provide descriptions of excluded volume in solution and generally do not attempt to model internal structure. However, the detection of flexibility and large volume-to-mass ratios in our analyses indicate that the +A RNP may also be somewhat distended in solution. We have previously reported on the unusual volume-to-mass determined by biophysical analysis of the ΔA particle, and suggested that the breadth and time-dependent properties of gel filtration and sedimentation velocity profiles might indicate a flexible species where multiple conformations are sampled in solution. Taken together with the results presented here, our studies indicate that the transition from the precursor to native state is accompanied by a significant size reduction and gain-of-order and compactness in the assembly. The driving force behind this structural transition, in the absence of ATP hydrolysis or other co-factors, is not yet understood.

Volumetric differences between the +A and ΔA particles may be due in part to the modest difference in composition (902 nt and 984 nt, respectively), although this discrepancy cannot entirely account for the large changes observed. It might be inferred from manual superposition of the +A and ΔA projections that these conformational changes correspond to RNA domain motions within the intron, as the lobes of volume which differ have elongated properties consistent with extended RNA domains. Indeed, previous proposals have suggested that domains II and VI mediate a conformational change that underlies self-splicing in both group IIA and IIB introns via the conserved η–η′ interaction (42,43,51). As domain II is known to enhance catalysis and domain VI contributes the catalytic adenosine residue, a structural model that couples this conformational change with the formation of a competent active site is plausible. In future studies, the methods employed here might provide the means to link the contributions of putative conserved tertiary interactions to overall quaternary structure of the *L. lactis* intron in different catalytic states, both with natively formed particles and materials prepared *in vitro*.

The shape of the +A particle determined by this study bears mechanistic and structural significance. First, our data indicate that the RNA component of the +A particle is distended by virtue of its flat ellipsoidal arrangement, but still more compact than the ΔA particle. It has been proposed that the binding of the LtrA protein to the LtrB RNA imparts a spatial organization that is required for RNA splicing (33). In the context of the model provided in this study, it can be envisioned that the high-affinity binding of the LtrA protein to the extended and exposed RNA region assigned as domain IV imparts significant structural ordering of the RNA component, priming the assembly for catalysis by bringing the distal globular RNA components into the proper spatial organization. The LtrA protein has also been shown to have secondary interactions with domains I, V and VI (18). Although SAXS lacks the spatial resolution to discern finer structural details such as secondary structure, the observed positioning of the protein phase in this reconstruction could readily accommodate additional contacts to the larger RNA lobes observed and hence would be compatible with these reported secondary interactions.

Our results also provide an opportunity to reconcile other proposed structural models with experimental solution properties. For example, previous studies of the intron-encoded LtrA protein by threading existing structures of HIV reverse transcriptase with LtrA sequence yielded a three-dimensional model that suggests RNA-binding surface (16). The spatial properties of the LtrA homology model dimer correlates well with the experimental SAXS envelope (Supplementary Figure S7). Manual docking of this structural model into the protein phase of the SAXS-derived envelope indicates that these models are generally compatible with the proposed interface, with a predicted binding surface near the center of mass.

Crystallographic structures of the related group IIC intron RNAs (22,23) are expected to be representative of the overall conserved secondary structure and gross spatial properties of the first five of the six structural domains of the intron RNA studied here. However, the composition of LtrB intron from *L. lactis* differs in mass by almost 3-fold, and the different subgroups of the group II introns vary significantly with respect to several predicted structural features. Hence, it is not expected that the available bacterial crystal structures can be directly reconciled with our experimental data, nor entirely account for its spatial properties. However, we note that the spatial arrangement of these structures do reveal a relatively planar organization of the structural elements of the intron.

An *ab initio* model derived from experimentally determined crosslinks accounting for 735 of the 902 nucleotides in the ΔORF RNP construct used in this study has also been reported (20). Although the missing nucleotides comprise a significant fraction of the overall inventory, the spatial extent and depth provided by this model can still be considered in light of our experimental results. Visual comparisons reveal that the solution properties of the intron RNA phase are substantially larger than those implied by the *ab initio* model (Supplementary Figure S7). Possible explanations for the apparent disparity between these structural models is the assumption that long range tertiary interactions previously identified in other self-splicing group II introns will be preserved (52,53) or that if they are maintained, that the interactions are transient.

The distinctive shape of the +A particle also bears significance with regards to macromolecular interactions. A consequence of anisotropic shapes is increased surface-to-volume (S/V) ratios. The axial ratios determined in this study for the +A particle indicate a particle volume much larger than would be predicted for a compact sphere of identical composition and as a consequence, a larger accessible surface. One possibility is that this elongated particle provides a lengthy interface for interaction of the intron with the DNA target over 14 base pairs (54). Another possibility relates to a large number of interacting host factors, including tRNA synthetases (11), DEAD box proteins (55,56), RNAses (57) and even the 30S ribosome (32). The structural significance of the oblate properties of the +A particle may lie in providing accessible interaction surfaces that are not mutually exclusive of other interactions and activities. The experimental approach outlined in this study provides a tractable strategy to probe the structural properties of these even-larger macromolecular assemblies alongside their biochemical properties, including hydrodynamic parameters, composition and stoichiometry. More broadly, such an integrative approach is applicable to the study of other model systems from which native assemblies can be obtained. Recent advances in synchrotron radiation applied to SAXS, detectors and microfluidics further this promise.

## SUPPLEMENTARY DATA

Supplemental Data are available at NAR Online. 

## FUNDING

The NSF & NIH/NIGMS via NSF award (DMR-0936384 to CHESS); NIGMS award (GM-103485 to MacCHESS); the US Department of Energy (DOE) (use of the Advanced Photon Source, an Office of Science User Facility operated for the US DOE Office of Science Argonne National Laboratory under Contract No. DE-AC02-06CH11357); the US DOE, Office of Science, Office of Basic Energy Sciences [Contract No. DE-AC02-98CH10886 for use of the National Synchrotron Light Source, Brookhaven National Laboratory]; the Welch Foundation (F-1756) and a DTRA Young Investigator Award (to L.M.C.); NIH grants [GM39422 and GM44844 to M.B.]. Funding for open access charge: Internal funds (Endowed Chair).

*Conflict of interest statement*. None declared.

## Supplementary Material

Supplementary Data
