# Supplementary material for: Quaternary arrangement of an active, native group II intron ribonucleoprotein complex revealed by small-angle X-ray scattering
Source: Nucleic Acids Res. 2014 Feb 24;42(8):5347–60. doi: 10.1093/nar/gku140 (PMC4005650; doi:10.1093/nar/gku140)
Supplement: Supplementary Data [file supp_42_8_5347__index.html]

Quaternary arrangement of an active, native group II intron ribonucleoprotein complex revealed by small-angle X-ray scattering — Quaternary arrangement of an active, native group II intron ribonucleoprotein complex revealed by small-angle X-ray scattering — Supplementary Data 

# Quaternary arrangement of an active, native group II intron ribonucleoprotein complex revealed by small-angle X-ray scattering

## Supplementary Data

files

**Files in this Data Supplement:**

- Supplementary Data - pdf file
