# Supplementary material for: Quaternary arrangement of an active, native group II intron ribonucleoprotein complex revealed by small-angle X-ray scattering
Source: Nucleic Acids Res. 2014 Feb 24;42(8):5347–60. doi: 10.1093/nar/gku140 (PMC4005650; doi:10.1093/nar/gku140)
Supplement: Supplementary Data [file supp_gku140_nar-03604-r-2013-File010.pdf]

Supplemental Materials

Quaternary Arrangement of an Active, Native Group II Intron Ribonucleoprotein Complex revealed by Small-angle X-ray Scattering

Kushol Gupta[1], Lydia M. Contreras[2,5], Dorie Smith[3], Guosheng Qu[3], Tao Huang[5,6], Lynn A. Spruce[4], Steven H. Seeholzer[1,4], Marlene Belfort[3,5], and Gregory D. Van Duyne[1]

[1]Department of Biochemistry & Biophysics, Perelman School of Medicine, University of Pennsylvania, Philadelphia, Pennsylvania, 19104-6059, U.S.A.

[2]Department of Chemical Engineering, University of Texas at Austin, Austin, Texas, 78712, U.S.A.

[3]Department of Biological Sciences and RNA Institute, University at Albany, State University of New York, Albany, NY, 12222, U.S.A.

[4]Children's Hospital of Philadelphia Research Institute, Philadelphia, Pennsylvania, 19104, U.S.A.

[5]Wadsworth Center, NYS Department of Health, Albany, NY 12201

[6]SUNY Downstate Medical Center, University Hospital, Brooklyn, NY 11203

Address correspondence to:

Kushol Gupta, Dept. of Biochemistry & Biophysics, Perelman School of Medicine, University of Pennsylvania, 810 Stellar-Chance Building, 422 Curie Boulevard, Philadelphia, PA 19104-6059
(Tel) 215-573-7260 (Fax) 215-573-4764. Email: kgupta@mail.med.upenn.edu

Supplemental Methods

Plasmid constructs. The sequences of all primers utilized in this study are listed in Supplemental Table 1. The 12xMS2 segment was amplified by the polymerase chain reaction (PCR) using pSL12xMS2 (31) as the template and primers IDT1080 and IDT1081, both of which encode flanking *NotI* restriction sites. The PCR product was ligated into the 3' end of exon2 of LtrB within linearized p+A/LtrA plasmid ((24), Supplemental Table 2) to create p+A(12MS2)/LtrA (Supplemental Table 2). This construct encodes the affinity-tagged nucleoprotein particle abbreviated as "+A" in this report. Of note, attempts to insert the 12xMS2 domain at the 5'exon yielded preparations with poor splicing activity relative to the 3'end tagged version employed.

To construct the domain II tRNA-containing intron, the LtrB ΔORF intron containing 15 bp of exon1 and 3 bp of exon2 was first cloned from pGEMΔORF (33) into the *XbaI* site of plasmid pBluescriptII SK(-) (pBSIISK$^-$, Stratagene, La Jolla, CA, U.S.A.), using primers IDT0058 and IDT0059. This pBSΔORF plasmid was then mutated with primer IDT0422 to introduce a *BglII* site in domain II of the intron using site-directed mutagenesis (QuickChange Kit, Stratagene, La Jolla, CA, U.S.A.). The tRNA (pheU) was amplified from *E. coli* with *BglII* ends using primers IDT0474 and IDT0475 and cloned into *BglII*-digested pBSΔORF to create pBSΔORF-DII-tRNA. The domain II-tRNA containing intron was then amplified by PCR with *NotI* and *XhoI* ends using primers IDT1264 and IDT1265 and cloned into the *NotI/XhoI* sites of p+A/LtrA to create p+A(DII(tRNA)/LtrA. Finally, the DII-tRNA-intron RNA sequence from this plasmid was amplified with *XhoI/AatII* ends with primers IDT1264 and IDT1265 and cloned into p+A(12MS2)/LtrA to form p+A(12MS2-DIItRNA)/LtrA, abbreviated to p+A-DII(tRNA).

In-solution digestion and mass spectrometry analysis. Samples were first treated with benzonase (Novagen) for 20 minutes at room temperature. Rapigest (Waters Corporation, Milford, MA, U.S.A.) acid labile surfactant was added to a final concentration of 0.1% before incubation at 50°C for 10 minutes followed by cooling to room temperature. Ten nanograms of trypsin (Promega Corporation, Madison, WI, U.S.A.) was then added and samples were incubated overnight at 37°C. Samples were then acidified with trifluoroacetic acid (TFA), incubated at 37°C for two hours, and spun in a table-top centrifuge to remove insoluble matter. Tryptic peptides were separated by reverse phase (RP)-HPLC on a nanocapillary column, 75 μ m id × 15 cm ChromXP 3µM 120Å (Eksigent Technologies LLC, Redwood City, CA, U.S.A.) in a Nanoflex chip system and analyzed with a hybrid LTQ Orbitrap Elite mass spectrometer (Thermofisher Scientific, Waltham, MA, U.S.A.). Mobile phase A consisted of 1% methanol/0.1% formic acid and mobile phase B of 1% methanol/0.1% formic acid/80% acetonitrile. Peptides were eluted into the mass spectrometer at 300 nL/min with each RP-LC run comprised of a 15 min. sample load at 3% B followed by a 90 minute gradient from 10 to 25% B and 25-40%B in 25 min. The mass spectrometer was set to repetitively scan m/z from 300 to 1800 (R = 240,000 for the LTQ-Orbitrap Elite) followed by data-dependent MS/MS scans on the twenty most abundant ions.

All MS/MS data were analyzed using Sorcerer SEQUEST (SageN) against a protein sequence database obtained from UNIPROT.org comprising a mixture of *E. coli*, *Lactococcus lactis lactis*, *Lactococcus lactis cremoris*, and common laboratory contaminants assuming the digestion enzyme semiTrypsin. SEQUEST was searched with a fragment ion mass tolerance of 1 Da and a precursor ion tolerance of 50 ppm. Oxidation of methionine was specified as a variable modification. Scaffold (version Scaffold_3.6.4, Proteome Software Inc.) was used to validate MS/MS based peptide and protein identifications using the Peptide- and Protein-Prophet algorithms (68,69) to establish a 1% false discovery rate.

Experimental Considerations for Small-Angle Scattering Analysis. Routine SAXS measurements on biological macromolecules <100 kD in mass typically demand >1 mg/mL sample concentrations for usable scattering information. The intensity of scatter from a particle can be expressed as such:

$$I(q) \propto \frac{N}{V} V^2_{particle} (\Delta\rho)^2 FF(q) S(q) \qquad (1)$$

Where $\frac{N}{V}$ is the number of particles per unit volume (concentration), $V_{particle}$ is the volume of the individual particle, Δρ is the contrast, FF(q), which is the form factor, or scattering component from a single particle rotationally averaged, and S(q), which is the interparticle structure factor.

Because of the relatively electron-rich nucleic acid component comprising the bulk of this particle's mass and the particle's relatively large volume (intensity of scattering varies as the square of particle volume), relatively low sample concentrations of RNP (0.05-0.1 mg/mL) provided measureable scatter at synchrotron sources. The added benefit of working with samples at these relatively dilute concentrations is the minimization of any potential interparticle interference (S(q)), which can undermine reliable shape reconstruction from the particle form factor. However, highly dilute samples demand a combination of low parasitic scatter and high detector sensitivity to obtain interpretable profiles. For large objects, intensity falls as q^{-4} in the wide angle (Porod) region. This situation results in rapid saturation of low-angle signals before wide-angle signals have accumulated enough counting statistics. The utilization of Pilatus counting detectors (Dectris Ltd., Baden, Switzerland) that feature high dynamic range combined with dual camera SAX/WAXS configurations proved particularly advantageous to these studies by addressing these concerns.

An important consideration made in this analysis is that the R_g and D_{max} values for protein-nucleic acid complexes determined by SAXS alone are generally depressed compared to values for a similar particle of uniform density, because the electron-dense nucleic acid component is localized to one end of the structure, relative to the center of mass of the entire complex. Theoretical contrast calculations using the program MuLCH (60) estimate contrasts (Δρ) of 2.8×10^{10} cm^{-2} and 5.8×10^{10} cm^{-2} for the protein and nucleic acid components of +A, respectively, in the buffer conditions used, indicating that the nucleic acid component is more strongly represented in the overall x-ray scattering. Hence, it is expected that our experimental data, in the absence of experimental contrast, is biased towards the RNA component of the RNP.

Small-Angle X-ray Scattering (SAXS) at CHESS Beamline F2 and G1. The particles examined in this study are only available in low yield (~ 1.5 μg/L of culture). The scarcity of sample material necessary for this study demanded minimal sample sizes for preliminary measurements and optimization of experimental parameters such as flux and exposure time. We implemented a strategy utilizing a 20 μl sample cell at the SAXS beam line at Cornell CHESS F2 and G1 (ALine, Redondo Beach, CA, USA) during multiple sessions for our preliminary synchrotron measurements, allowing us to rigorously examine experimental variables such as flux and exposure time while minimizing sample consumption. Samples were either centrifuged at 10,000xg for 2-5 min or filtered through a 0.45 μm filter at 4°C before exposures ranging from 5s to 60s were recorded at 4°C. The forward scattering from the samples studied was recorded on a CCD detector and circularly averaged using the program Data Squeeze 2.07 (Datasqueeze Software, Wayne, P.A., USA) or the program BioXTAS RAW (61) to yield one-dimensional intensity profiles as a function of q ($q=4\pi\sin\theta/\lambda$, where 2θ is the scattering angle). Scattering from a matching buffer solution was subtracted from the data, and data were corrected for the incident intensity of x-rays. Replicate exposures were examined carefully for evidence of radiation damage by Guinier analysis and Kratky plot analysis before averaging and merging. Silver behenate powder was used to locate the beam center and to calibrate the sample-to-detector distance. Intensity data were converted to absolute units (cm^{-1}) using the scatter from water.

Small-angle x-ray scattering at the Advanced Photon Source beam line 18-ID (BIOCAT). X-ray radiation at a wavelength of 0.968 Å was used to collect 10-15 individual one second exposures while the sample was oscillated in a quartz capillary to minimize radiation damage. Data were corrected for the intensity of the incident radiation and reduced to provide one-dimensional intensity profiles as a function of q using the data software Igor Pro (WaveMetrics, Lake Oswego, O.R., U.S.A.). Accessible scattering

was recorded in the range of $0.006 < q < 0.35$ Å^{-1} (where q=4πsinθ/λ, where 2θ is the scattering angle). Exposures were examined for radiation damage before averaging and subsequent subtraction of buffer scatter. Samples were 0.45 µm filtered by centrifugation at 4°C through a Millipore spin filter (Millipore Corp., Bedford, M.A., U.S.A.). Measurements were made in a quartz capillary using a 60 µL loading program with oscillating flow. Fifteen 1-second exposures at 4°C were recorded with 8 x 20 mm aluminum foil attenuation and corrected for incident intensity. Individual scattering profiles were examined for evidence of radiation damage before averaging and subtraction of buffer profile.

Small-angle x-ray scattering at the National Synchrotron Light Source beam line X9 (NSLS). Scattering profiles collected at X9 were collected with a MarCCD SAXS detector and a Photonic Science WAXS detector. Measurements were taken at room temperature with a sample to detector distances of D/d = 20.3 for SAXS and 4.67 for WAXS. The x-ray wavelength used was 0.855λ. The scattering profiles covered a range of $0.01 < q < 2.0$ Å^{-1} (where q is equal to 4πsinθ/λ, where 2θ is the scattering angle), but data was only used to $q < 0.50$ Å^{-1} for subsequent analyses. The sample holder was a 1 mm quartz capillary (Hampton Research, Aliso Viejo, C.A., U.S.A.) that was sealed across the evacuated beam path. The capillary was open at both ends to allow a continuous flow of the sample to minimize radiation damage. Samples were 0.45 µm filtered by centrifugation at 4°C through a Millipore spin filter. Three measurements of 30s-90s exposure times were performed on a 30 µl sample and examined for evidence of radiation damage before averaging.

SAXS Data Analysis. All of the preparations analyzed were monodisperse, as evidenced by linearity in the Guinier region of the scattering data and agreement of the I(0) and the radius of gyration (R_g) values determined with inverse Fourier transform analysis by the program GNOM (45). When fitting manually, the maximum diameter of the particle (D_{max}) was adjusted in 5-10 Å increments in GNOM to maximize the goodness-of-fit parameter, to minimize the discrepancy between the fit and the experimental data, and to optimize the visual qualities of the distribution profile. Shape fitting to the primary data and using known form factors was performed using SASFit (https://kur.web.psi.ch/sans1/SANSSoft/sasfit.html).

Shape reconstruction. Low resolution shapes were determined from solution scattering data using either the programs MONSA or DAMMIF, from the ATSAS suite of programs (45). Bead models were visualized in PYMOL (Version 1.5.0.4 Schrödinger, LLC.). For MONSA calculations, the search space was constrained to the ellipsoid determined by form factor analysis and phase volumes restrained to the volume calculated for each phase based on composition. Ten independent calculations were performed with no symmetry constraints, and the final results were superposed and averaged using MONSAVER, a modified version of the DAMAVER program suite (45).

For DAMMIF calculations, thirty independent calculations were performed for each data set using default parameters and no symmetry constraints. The models resulting from the independent runs were superimposed by the program DAMSUP based on normalized spatial discrepancy (NSD) criterion. The thirty independent reconstructions were then averaged and filtered to a final consensus model using the DAMAVER suite of programs (45). Bead models generated by DAMMIF were converted to volumetric representations using the program SITUS (63) and visualized using UCSF Chimera (64). The programs HYDROPRO (65) and SOLPRO (66) were used to calculate hydrodynamic properties of experimental cryo-EM or SAXS reconstructions. Particle dimensions were determined using the *pdb2vol* application of the SITUS program suite.

References.

58. Eng, J., McCormack, A. and Yates, J. (1994) An approach to correlate tandem mass spectral data

of peptides with amino acid sequences in a protein database. *Journal of The American Society for Mass Spectrometry*, **5**, 976-989.

59. Perkins, D.N., Pappin, D.J., Creasy, D.M. and Cottrell, J.S. (1999) Probability-based protein identification by searching sequence databases using mass spectrometry data. *Electrophoresis*, **20**, 3551-3567.
60. Whitten, A.E., Cai, S. and Trewhella, J. (2008) MULCh: Modules for the analysis of small-angle neutron contrast variation data from biomolecular assemblies. *J. Appl. Crystallogr.*, **41**, 222-226.
61. Nielsen, S.S., Toft, K.N., Snakenborg, D., Jeppesen, M.G., Jacobsen, J.K., Vestergaard, B., Kutter, J.P. and Arleth, L. (2009) BioXTAS RAW, a software program for high-throughput automated small-angle X-ray scattering data reduction and preliminary analysis. *J. Appl. Crystallogr.*, **42**, 959-964.
62. Rambo, R.P. & Tainer, J.A. Characterizing flexible and intrinsically unstructured biological macromolecules by SAS using the Porod-Debye law. *Biopolymers* **95**, 559-571 (2011).
63. Wriggers, W., Milligan, R.A. and McCammon, J.A. (1999) Situs: A package for docking crystal structures into low-resolution maps from electron microscopy. *J. Struc. Biol.*, **125**, 185-195.
64. Pettersen, E.F., Goddard, T.D., Huang, C.C., Couch, G.S., Greenblatt, D.M., Meng, E.C. and Ferrin, T.E. (2004) UCSF chimera - A visualization system for exploratory research and analysis. *J Comp. Chem.*, **25**, 1605-1612.
65. Garcia de la Torre, J., Huertas, M.L. and Carrasco, B. (2000) Calculation of hydrodynamic properties of globular proteins from their atomic-level structure. *Biophys. J.*, **78**, 719-730.
66. Garcia de la Torre, J., Carrasco, B. and Harding, S.E. (1997) SOLPRO: theory and computer program for the prediction of SOLution PROperties of rigid macromolecules and bioparticles. *European Biophysics Journal*, **25**, 361-372.
67. Voss, N.R. and Gerstein, M. (2005) Calculation of standard atomic volumes for RNA and comparison with proteins: RNA is packed more tightly. *J. Mol. Biol.*, **346**, 477-492.
68. Keller, A., Nesvizhskii, A.I., Kolker, E. and Aebersold, R. (2002) Empirical statistical model to estimate the accuracy of peptide identifications made by MS/MS and database search. *Analytical chemistry*, **74**, 5383-5392.
69. Nesvizhskii, A.I., Keller, A., Kolker, E. and Aebersold, R. (2003) A statistical model for identifying proteins by tandem mass spectrometry. *Analytical chemistry*, **75**, 4646-4658.

A.

MKPTMAILERISK**NSQENIDEVFTR**LYRYLLRPDIYYVAYQNLYSNKGASTK**GILDDTAD**
GFSEEKIKK**IIQSLKDGTYYPQPVRR**MYIAKKNSKK**MRPLGIPTFTDK**LIQEAVRIILES
IYEPVFEDVSHGFRPQRSCHTALKTIKREFGGAR**WFVEGDIK**GCFDNIDHVTLIGLINLK
IKDMK**MSQLIYKFLK**AGYLENWQYHKTYSGTPQGGILSPLLANIYLHELDKFVLQLKMKF
DRESPERITPEYR**ELHNEIKR**ISHRLKKLEGEEKAK**VLLEYQEKR**KRLPTLPCTSQTNKV
LKYVR**YADDFIISVK**GSKEDCQWIKEQLKLFIHNK**LKMELSEEKTLITHSSQPARFLGYD**
IRVRRSGTIKRSGKVKKR**TLNGSVELLIPLQDK**IRQFIFDKKIAIQKKDSSWFPVHRKYL
IR**STDLEIITIYNSELR**GICNYYGLASNFNQLNYFAYLMEYSCLKTIASKHKGTLSKTIS
MFK**DGSGSWGIPYEIK**QGKQRRYFANFSECKSPYQFTDEISQAPVLYGYARNTLENRLKA
KCCELCGTSDENTSYEIHHVNKVKNLKGK**EKWEMAMIAK**QRKTLVVCFHCHRHVIHKHK

B.

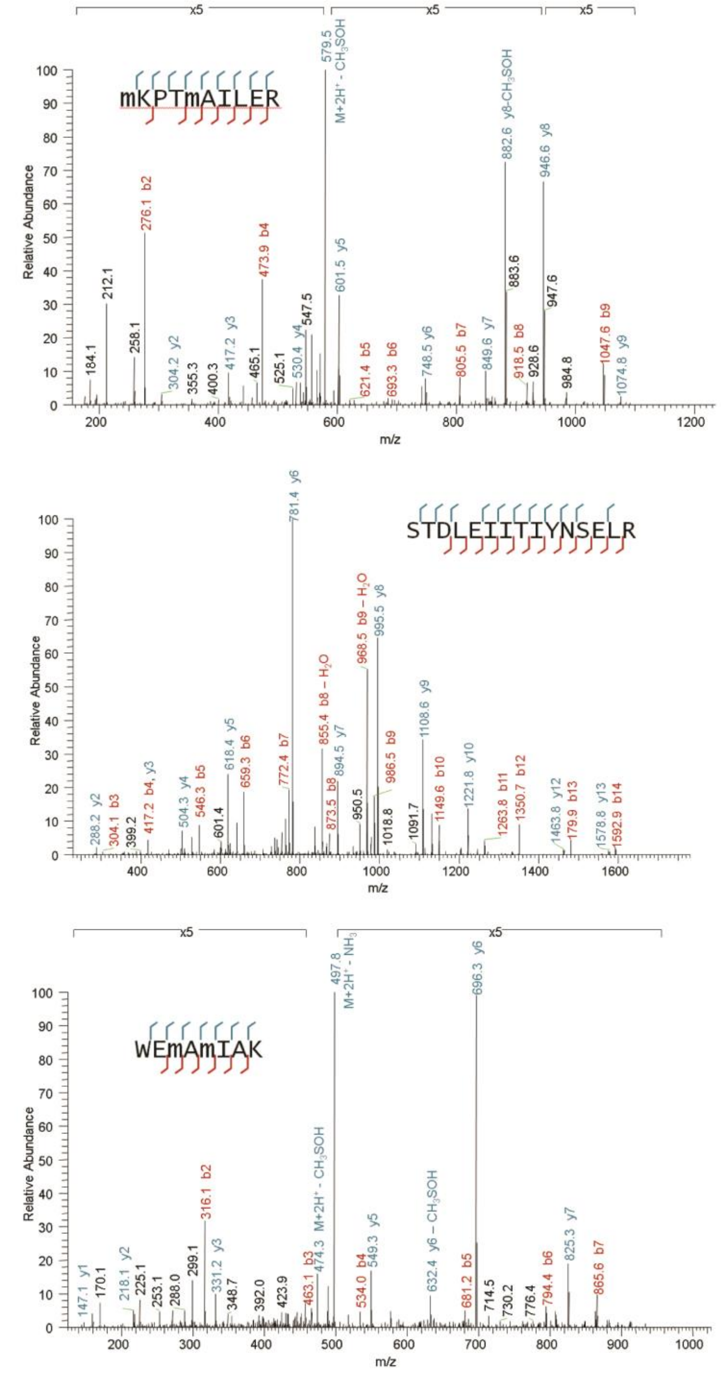

Supplemental Figure 1. A. Primary sequence of *L. Lactis* LtrA (Accession code D8KIX0). Highlighted in magenta are the segments of the sequence identified from LC-MS-MS analysis of the purified intron particle. B. Identification of LtrA in native preparations of Group II intron-derived particles from *L. lactis*. Shown are three representative tandem mass spectrum derived from peptides at the N-terminus (upper), internal (middle), and near the C-terminus (lower) of the LtrA protein. Shown are the spectra of the precursor ion and the corresponding peptide sequence predicted by MASCOT (59), showing b- and y-type ions. Amino acid sequence of the complete ORF identified by 15 unique tryptic peptides within Y unique spectra; the identified peptides are underlined and comprise 35% of the total sequence. Hash marks shown in the respective sequences indicate b- and y-ions assignments. Neutral loss of elements of CH_3SOH from Met-oxide-containing ions is indicated. Met-oxide is represented by lower case m in sequences. Met oxidation is likely to be an artifact of proteomic analysis. Of note, this approach as implemented can only verify the presence of a macromolecule in detectable amounts and not determine its absolute quantity, and hence, stoichiometry.

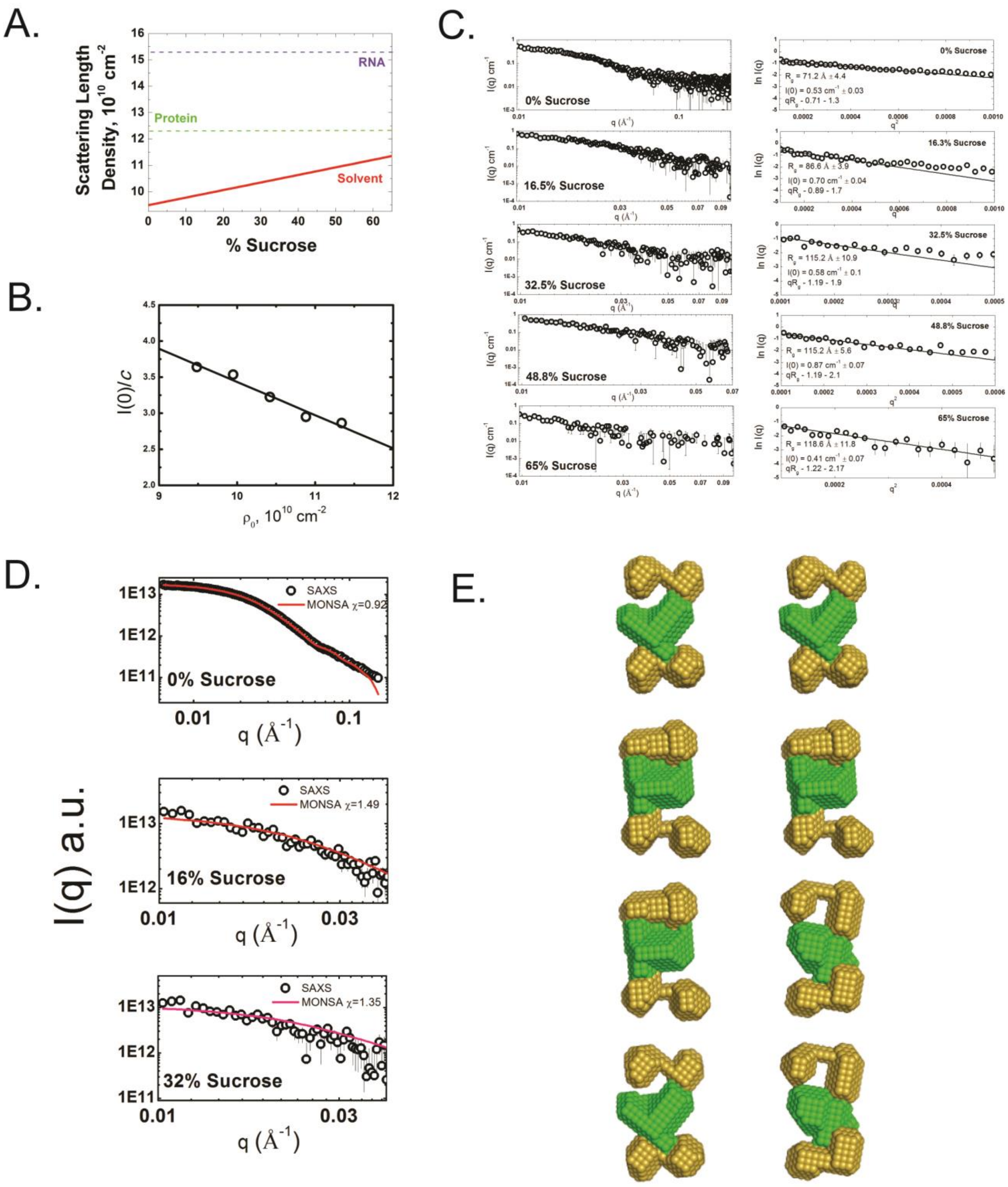

Supplemental Figure 2. Contrast Variation Analysis. A. Calculated scattering length densities of the protein and RNA components of the +A particle as a function of sucrose concentration (%) in the buffer used in this study. B. A linear dependence of the intensity of scattering normalized for concentration (I(0)/c) as a function of contrast is observed as adjusted by sucrose. C. Experimental intensity profiles

(left) and Guinier analyses (right) for +A in increasing concentrations of sucrose. Data were recorded at Cornell CHESS F2. The recorded intensity is shown as a function of q ($q=4\pi\sin\theta/\lambda$, where 2θ is the scattering angle). Parameters derived from this analysis are summarized in Table 1. Error bars represent plus and minus the combined standard uncertainty of the data collection. D. Representative MONSA fits (red) to experimental data (open black circles). E. Representative gallery view of MONSA reconstructions using three experimental SAXS contrast points. Sphere size equals 5.75 Å.

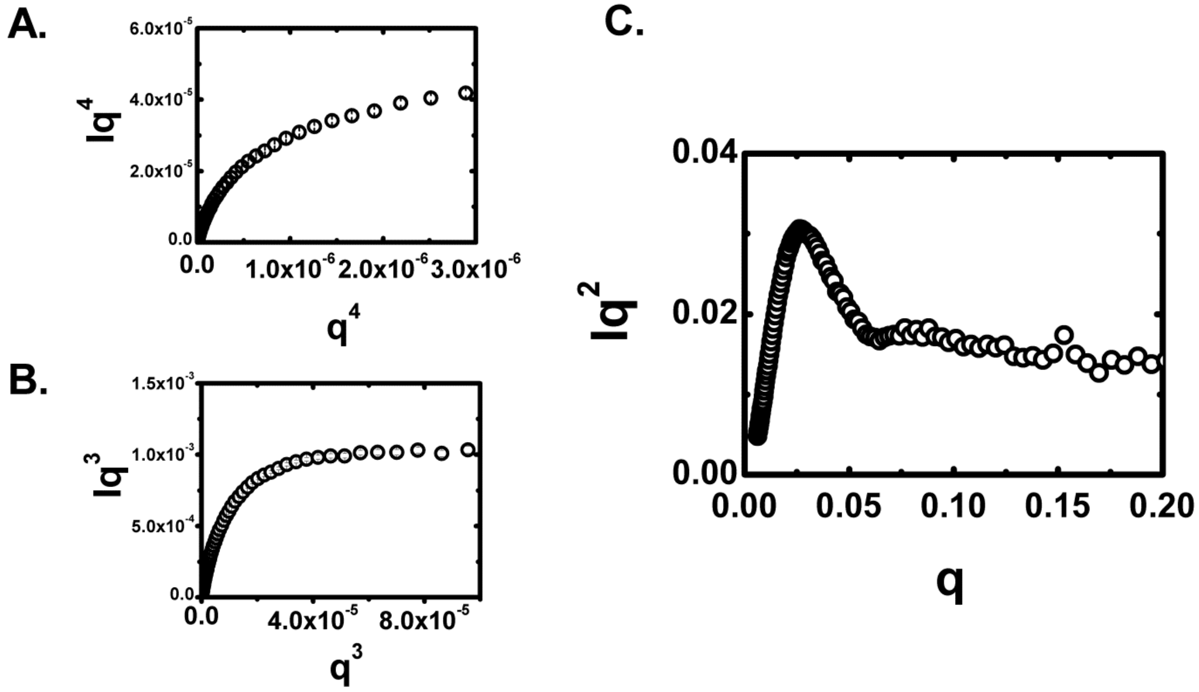

Supplemental Figure 3. Evidence for intrinsic flexibility in the native +A particle. A&B. Porod-Debye analysis (62). A plateau in the profile as a function of q^4 would indicate overall compactness, whereas deviation from this asymptote (as seen in **A**) as a function of q^4 is proportional to the loss of compactness. The data for +A is better described by a q^3 behavior (**B**), indicating a particle with a mixture of flexible and folded components. **C.** Kratky plot analysis provides a qualitative assessment of the degree of compaction of a biopolymer. For a well-folded macromolecule, a distinct peak feature at low scattering angle is typically observed, followed by a return to baseline. Fully-unstructured biopolymers lack these canonical features, instead increasing systematically with scattering angle. In this analysis, elements of both flexibility and ordered structure are indicated.

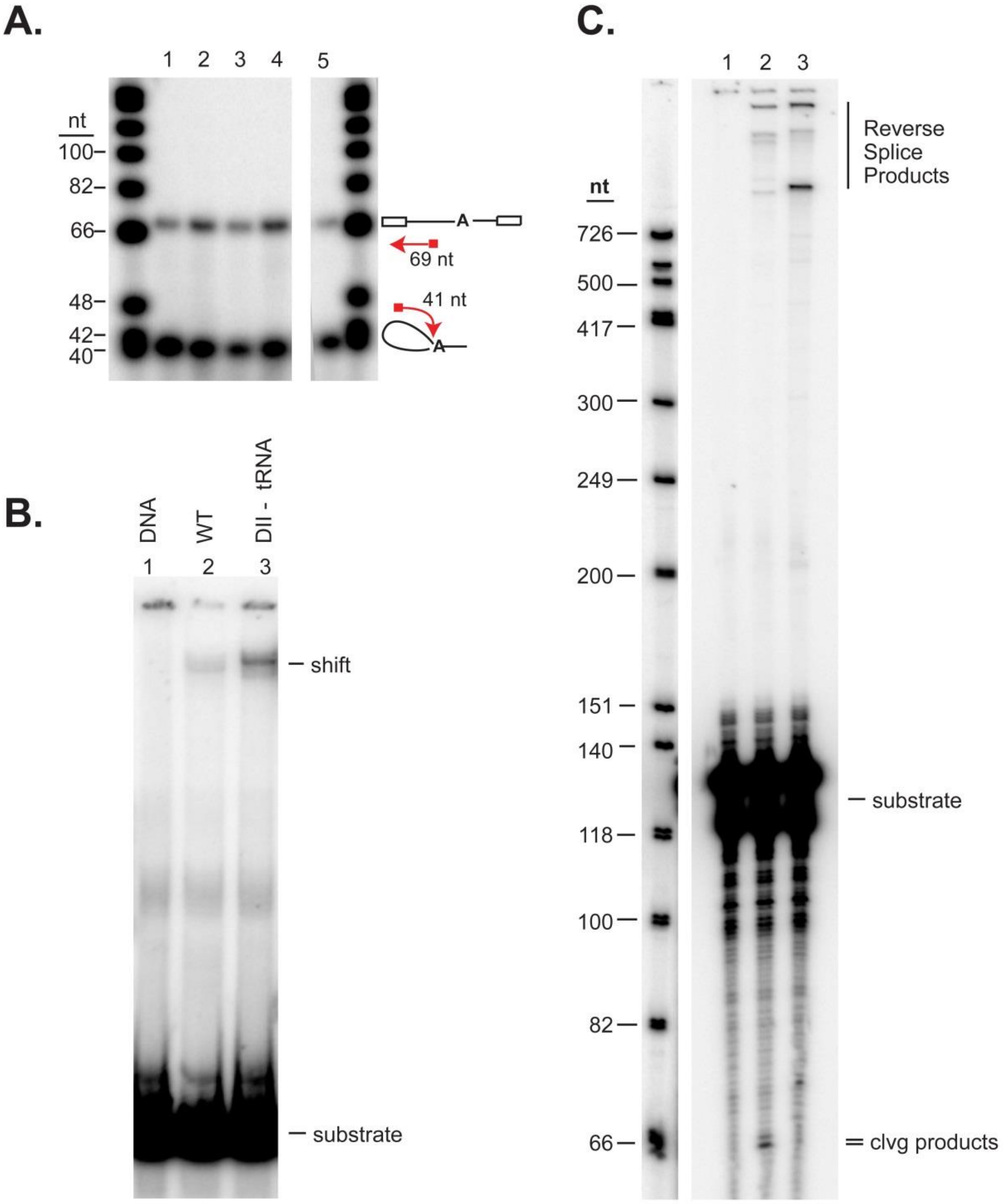

Supplemental Figure 4. The +A-DII (tRNA) construct is active. A. Splicing activity assay. Primer extension was performed as in Figure 2C. Lanes 1-4 contain tRNA fusions in domains I, II, IV and VI, respectively, whereas lane 5 contains the parental control without tRNA. **B.** DNA binding assay. The electrophoretic mobility shift assay (32) showed that the +A-DII (tRNA) (lane 3) construct bound the DNA target at least as well as the wild-type counterpart (lane 2). **C.** Reverse splicing assay. The assay, as in Figure 3B & C, shows that although bottom-strand cleavage is compromised in the +A-DII(tRNA) mutant, reverse splicing is more robust in the mutant (lane 2) than the parental +A construct (lane 3).

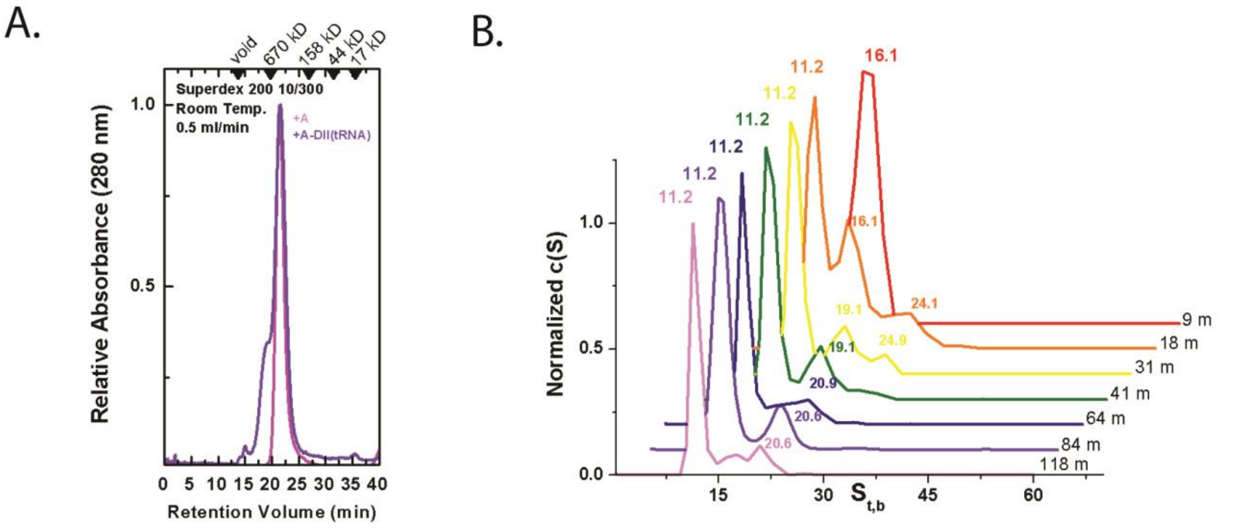

Supplemental Figure 5. A. +A-DII(tRNA) is unstable at room temperature. Gel filtration chromatogram comparing the elution profiles of +A (pink) and +A-DII(tRNA) (purple). While the +A shows evidence of remaining intact during the course of the experiment at room temperature, the +A-DII(tRNA) shows evidence of polydispersity not seen by in the initial boundaries by sedimentation velocity at 4°C. Hence, for our SAXS experiments, the material was maintained at low temperature during data collection. **B. Pressure-induced dissociation of +A-DII(tRNA) in analytical centrifugation**. Shown are c(S) profiles determined by fitting of the Lamm equation to five data boundaries across the different time ranges denoted to the right, within the centrifugation experiment performed at 4°C and 25,000 RPM. While the particle initially appears as a single ~16S species in the first 10 minutes of the experiment (see Figure 4), it progressively dissociates to yield ~20S and ~11S species over the course of two hours. This dissociative behavior is not observed with the native +A particle. The pressure in the cell, as calculated by SEDFIT, is 21 bar in the middle of the cell and 43 bar at the bottom.

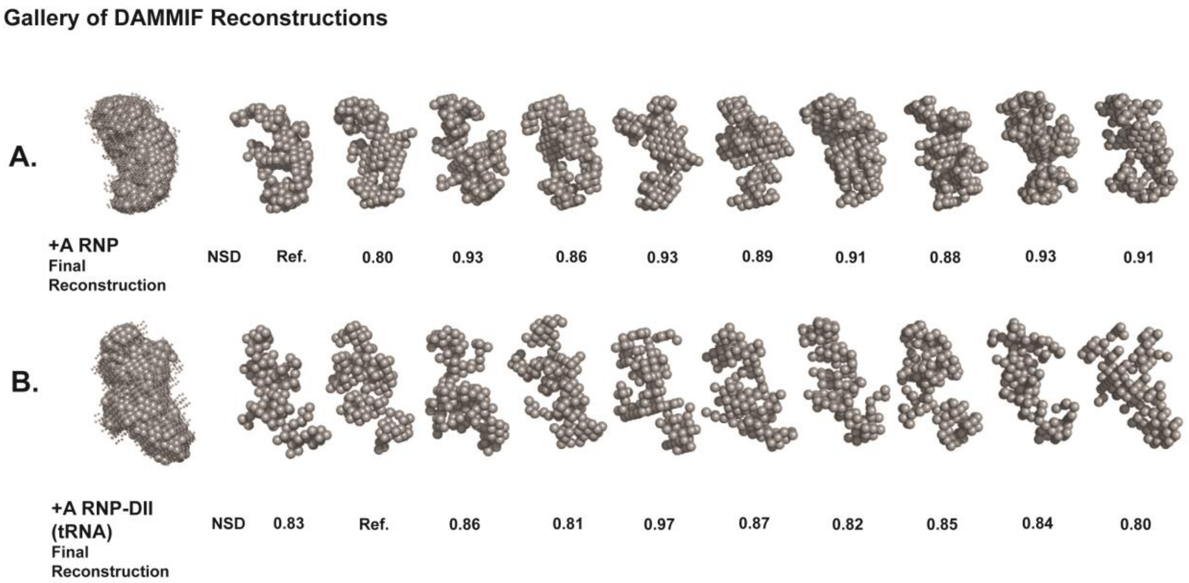

Supplemental Figure 6. Representative Gallery of DAMMIF shape reconstructions. The final averaged and filtered reconstructions, and their corresponding galleries of individual reconstructions are shown for +A RNP alone (A) and the +A RNP variant with the DII tRNA insertion (B). These reconstructions were calculated by the program DAMMIF and rendered as bead models with a sphere radius of 6.25 Å and 7.0 Å, respectively. The normalized spatial discrepancy (NSD) figure quantitates the similarity between two bead models; a low value corresponds to good overlap, with a value of one generally indicating high similarity. The shape calculations shown incorporate no symmetry constraints and yielded consistent results, with χ^2s at or near 1.0.

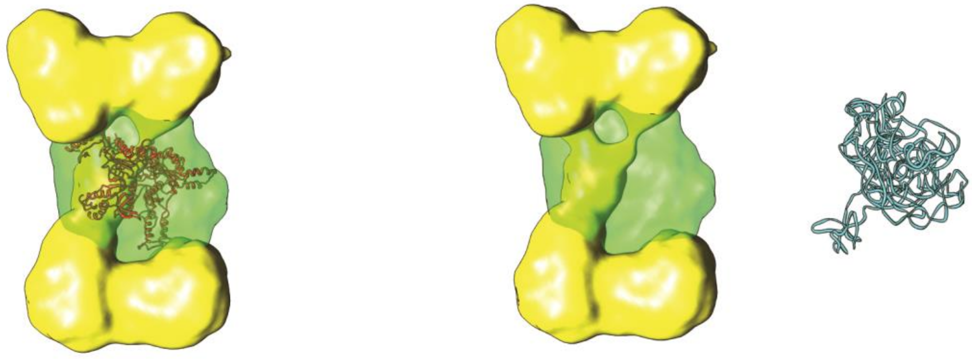

Supplemental Figure 7. Comparison with existing *L. lactis* Intron Models. Volumetric Representations of final MONSA two-phase models are shown. Green represents the protein phase and yellow the RNA phase. On the left, the protein phase is docked with the dimer LtrA homology model (red, (16)), showing good spatial correlation. On the right, the *ab-initio* Intron RNA model previously proposed (blue,(20)) is shown adjacent to the same the SAXS-derived reconstruction. The volumetric envelopes were created using SITUS (63) and rendered using UCSF Chimera (64).

Supplemental Table 1. Oligonucleotides used this in study.

Primer	Sequence
IDT1080	5'-AAG GAA AAA AGC GGC CGC GAT TAC GAA TTC GAA TGG CCA TGG G -3'
IDT1081	5'-TTT TCC TTT TGC GGC CGC CCT GCA GGG CAT CCT AGG CCT ATT AAT ATT CCG-3'
IDT0422	5'-GGA GGA AAA AGG CTA TAG ATC TAG AGC TTG AAA ATC TTG 3'
IDT0474	5'-GCG AGA TCT CCC GGA TAG CTC AGT CGG TA 3'
IDT0475	5'-GCG AGA TCT TTG GTG CCC GGA CTC GGA AT 3'
IDT1073	5'-GTA CCT TAA ACT ACT TGA CTT AAC ACC -3'
IDT0058	5'-CGC GGA TCC GAA CAC ATC CAT AAC GTG CGC CCA GAT AGG GTG TTA-3'
IDT0059	5'-CGC GGA TCC ATG GTG AAG TAG GGA GGT ACC GCC TTG-3'
IDT1264	5'-CTC GAG TCT AGA GAA CAC ATC CAT AAC GTG CGC-3'
IDT1265	5'-GAC GTC GCG GCC GCA GAA TTA AAA ATG ATA TGG TGA AGT AGG GAG GTA C-3'

Supplemental Table 2. Plasmids used this in study.

Plasmid Name	Origination annotation	Ref.
p+A/LtrA	pLNRK-nisLtrB(ΔORF+A)+nisLtrA-Intein-Chitin Binding Domain (CBD) plasmid	(24)
p+A(12MS2)/LtrA	pLNRK-nisLtrB(ΔORF+A)-12MS2-E2+nisLtrA-Intein-CBD	This work
pBSΔORF	LtrB ΔORF intron containing 15 bp of Exon1 and 3 bp of Exon 2 was first cloned into the *XbaI* site of plasmid pBSIISK$^-$	(24)
pBSΔORF DII-tRNA	tRNA (pheU) was amplified from *E. coli* with *BglII* ends using primers IDT0474 and IDT0475 and cloned into *BglII*-digested pBSΔORF	This work
p+A(DIItRNA)/LtrA	pLNRK-nisLtrB(ΔORF+A-DII-(tRNA))+nisLtrA-Intein-Chitin Binding Domain (CBD) plasmid	This work
p+A(12MS2-DIItRNA)/LtrA	pLNRK-nisLtrB(ΔORF+A-DII-(tRNA))-12MS2-E2+nisLtrA-Intein-CBD	This work

Supplemental Table 3. Table of Calculated Properties of particles examined in this study and their component parts.[a]

	Residues	Volume (A^3)	Molecular Weight (Daltons)	Partial Specific Volume (mL/g)
Components				
tRNA	76 n.t.	23,173.9	24,515.2	0.5693
+A RNA	902 n.t.	275,792.0	291,271.4	0.5702
ΔA RNA	984 n.t.	300,645.8	317,609.8	0.5700
LtrA Protein Dimer	1,198 a.a.	174,477.7	140,297.2	0.7489
Complexes				
+A RNP	1,198 a.a. + 902 n.t.	450,269.7	431,568.6	
ΔA RNP	1,198 a.a. + 984 n.t.	475,123.5	457,907.0	
+A RNP/DII tRNA	1,198 a.a. + 978 n.t.	473,443.6	456,083.8	

[a]as calculated by NucProt (67) based on chemical composition.
